# Tidally modified western boundary current drives interbasin exchange between the Sea of Okhotsk and the North Pacific

**DOI:** 10.1038/s41598-021-91412-y

**Published:** 2021-06-08

**Authors:** Hung-Wei Shu, Humio Mitsudera, Kaihe Yamazaki, Tomohiro Nakamura, Takao Kawasaki, Takuya Nakanowatari, Hatsumi Nishikawa, Hideharu Sasaki

**Affiliations:** 1 Pan-Okhotsk Research Center, Institute of Low-Temperature Science, Hokkaido University, Sapporo, 060-0819 Japan; 2 Graduate School of Environmental Science, Hokkaido University, Sapporo, 060-0810 Japan; 3 Institute of Low-Temperature Science, Hokkaido University, Sapporo, 060-0819 Japan; 4 Atmosphere and Ocean Research Institute, The University of Tokyo, Kashiwa, 277-8564 Japan; 5 Fisheries Resource Institute, Japan Fisheries Research and Education Agency, Kushiro, 085-0802 Japan; 6 Application Laboratory, Japan Agency for Marine-Earth Science and Technology, Yokohama, 236-0001 Japan

**Keywords:** Physical oceanography, Fluid dynamics

## Abstract

The interbasin exchange between the Sea of Okhotsk and the North Pacific governs the intermediate water ventilation and fertilization of the nutrient-rich subpolar Pacific, and thus has an enormous influence on the North Pacific. However, the mechanism of this exchange is puzzling; current studies have not explained how the western boundary current (WBC) of the subarctic North Pacific intrudes only partially into the Sea of Okhotsk. High-resolution models often exhibit unrealistically small exchanges, as the WBC overshoots passing by deep straits and does not induce exchange flows. Therefore, partial intrusion cannot be solely explained by large-scale, wind-driven circulation. Here, we demonstrate that tidal forcing is the missing mechanism that drives the exchange by steering the WBC pathway. Upstream of the deep straits, tidally-generated topographically trapped waves over a bank lead to cross-slope upwelling. This upwelling enhances bottom pressure, thereby steering the WBC pathway toward the deep straits. The upwelling is identified as the source of joint-effect-of-baroclinicity-and-relief (JEBAR) in the potential vorticity equation, which is caused by tidal oscillation instead of tidally-enhanced vertical mixing. The WBC then hits the island chain and induces exchange flows. This tidal control of WBC pathways is applicable on subpolar and polar regions globally.

## Introduction

The water exchange between the Sea of Okhotsk and the North Pacific is an essential component of the overturning circulation that ventilates the intermediate layer of the North Pacific^1^. Surface water originating in the subarctic gyre enters the Sea of Okhotsk through the Kuril straits^1, 2^. It then subducts to the intermediate layer (300–500 m, within the 1026.8–1027.0 kg m^−3^ isopycnals) due to active sea ice formation over the northern continental shelves^1, 2^, and finally outflows to the North Pacific across the Kuril straits, resulting in the North Pacific Intermediate Water (NPIW)^1, 2^ that circulates in the subtropical gyre. Further, the outflow from the Sea of Okhotsk entrains abundant nutrient materials such as iron, which support high primary production contributing not only to abundant fisheries resources but also to a vast amount of CO_2_ uptake in the western North Pacific^3–5^.

**Figure 1:**
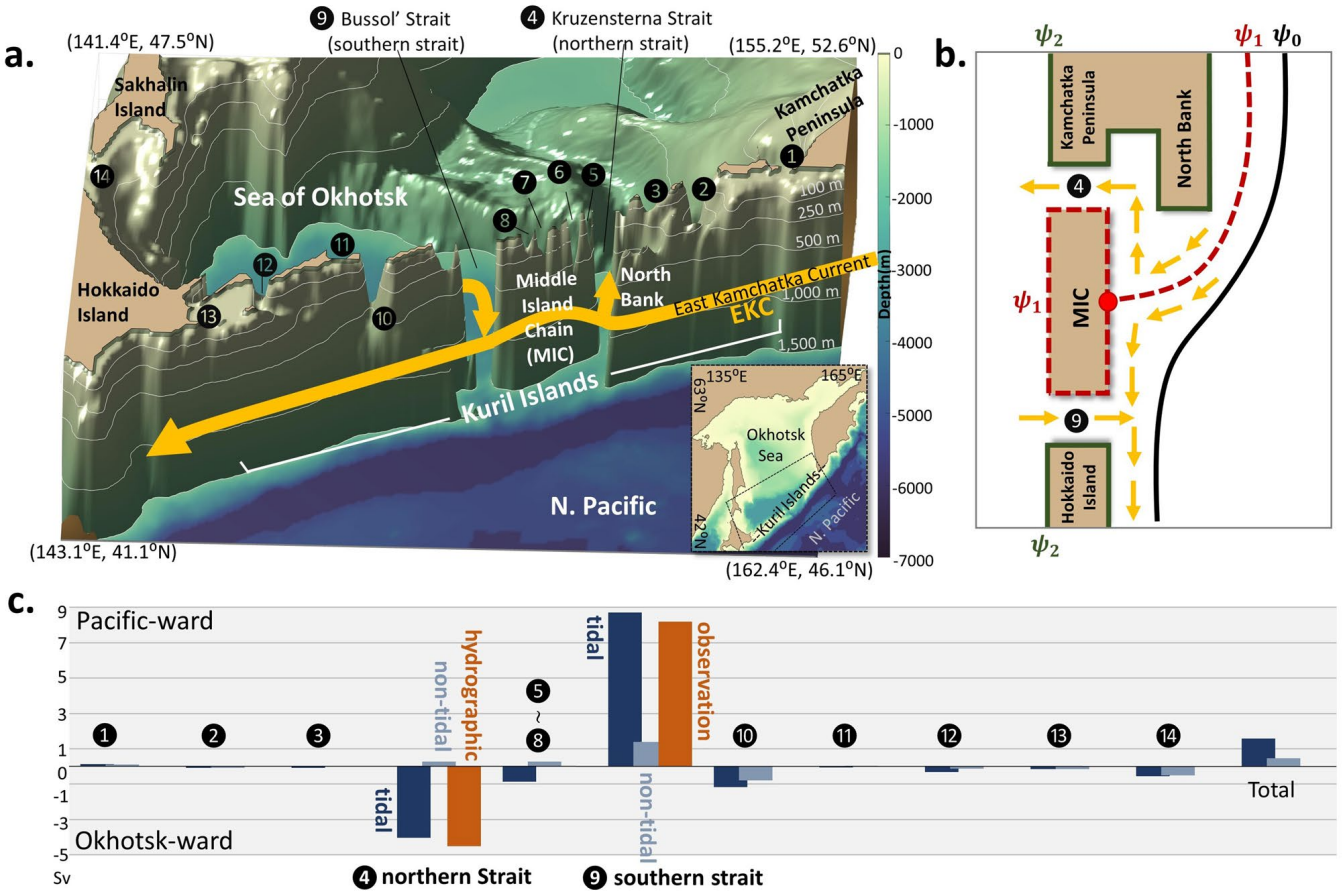
Okhotsk-Pacific Exchange System through different straits. (**a**) Topographic features of the Kuril Islands in the COCO model, which is adapted from the Japan Oceanographic Data Center (JODC) and modified by Ono et al.^35^ and Matsuda et al.^15^ (see the “Methods” section). The shaded areas represent the depth of the ocean and contours denote the iso-depths of 100, 250, 500, 1000, and 1500 m. To illustrate the details of the strait topographic features, the bird’s-eye view plot extends only to a depth of 1500 m, whereas the rest of the study area is represented as shades on the floor. The arrows represent the East Kamchatka Current (EKC), the western boundary current, and the major exchange currents through the Kruzensterna and Bussol’ Straits. The circled numbers above straits denote the straits from north to south as follows: 1. 1st Kuril Strait, 2. Onekotan Strait, 3. Shiashikotan Strait, 4. Kruzensterna Strait (northern strait), 5. Matua Strait, 6. Rasshua Strait, 7. Ketoy Strait, 8. Simushir Strait, 9. Bussol’ Strait (southern strait), 10. Urup Strait, 11. Etrof Strait, 12. Kunashiri Strait, 13. Nemuro Strait and 14. Soya Strait. The abbreviation “N. Pacific” stands for North Pacific. All these strait numbers are used in figures throughout this study. (**b**) Schematic view of the Okhotsk-Pacific exchange system by streamfunction. The black and army green solid line denotes the streamfunction ${\psi }_{0}$ as the offshore boundary of the EKC and the streamfunction ${\psi }_{2}$ as the boundary around the Kamchatka Peninsula, as well as Hokkaido Island, respectively. The red dashed line represents the streamfunction ${\psi }_{1}$ as the core axes of the EKC and the streamfunction around the MIC. The red circle indicates the bifurcation point of the EKC on the MIC. The EKC flows from the North Bank to the MIC by following the streamfunction contour ${\psi }_{1}$ as the main axes with ${\psi }_{0}$ as the offshore boundary of the EKC. When the EKC approaches the MIC, it collides on the streamfunction contour surrounding the MIC and forms the bifurcation point. The transport at the northern and southern straits are thus ${\psi }_{2}-{\psi }_{1}$ and ${\psi }_{1}-{\psi }_{2}$, respectively. (**c**) Annual-averaged volume transport through each strait, where the numbers in the horizontal axis correspond to the numbers in (**a**). Positive values denote a Pacific-ward flow, whereas negative values are Okhotsk-ward. The tidal case, the non-tidal case, and observation data^6–8^ are indicated in navy-blue, grey-blue, and dark-orange bars, respectively.

Despite this significance, the mechanisms that drive the Okhotsk-Pacific exchange remain largely uncharacterized, as the East Kamchatka Current (EKC; see Fig. 1a), a western boundary current (WBC) of the subarctic North Pacific, intrudes only partially into the Sea of Okhotsk through straits along the Kuril Islands. Current studies have not yet elucidated what determines the EKC’s partial intrusion. Most of the interbasin exchange occurs through the two deepest straits along the Kuril Islands^6–8^, which are the Kruzensterna Strait to the north (hereinafter referred to as ‘the northern strait’; No. 4 in Fig. 1a with a depth of 1900 m^8^) and the Bussol’ Strait to the south (hereinafter referred to as ‘the southern strait’; No. 9 in Fig. 1a with a depth of 2200 m^6, 9^), separated by the Middle Island Chain (MIC). Based on hydrographic data, Katsumata and Yasuda^7^ published a concise summary of previous throughflow estimates, in addition to their estimate of − 5.6 Sv (1 Sv $\equiv {10}^{6}$ m^3^ s^−1^) and + 6.6 Sv (annual average) through the northern and southern straits, respectively, where the negative (positive) symbol denotes the Okhotsk-ward (Pacific-ward) throughflow. Further, based on lowered acoustic Doppler current profiler measurements, Katsumata et al.^6^ estimated an outflow of approximately + 8.2 to + 8.8 Sv via the southern strait during summer. Although transport estimates are rare, the exchange likely occurs with an Okhotsk-ward inflow through the northern strait and a Pacific-ward outflow through the southern strait^7^. Kida and Qiu^10^ reported that the intrusion of the EKC (Fig. 1b) would occur when the EKC collided with the MIC after passing the North Bank upstream (Fig. 1a; 86 m depth at the top^11^) by creating a bifurcation (red dot in Fig. 1b) on a streamfunction contour ${\psi }_{1}$ that coincides with the contour surrounding the MIC. A part of the EKC branches northward at the bifurcation and forms a partial intrusion with a transport of ${\psi }_{2}- {\psi }_{1}$ (Fig. 1b), where ${\psi }_{2}$ is the streamfunction contour along the land on the other side of the MIC. Therefore, the partial exchange occurs due to the presence of the EKC’s bifurcation on the MIC.

A question has arisen regarding the processes that control the bifurcation that leads to the partial intrusion. Kida and Qiu^10^ evaluated ${\psi }_{1}$ around an island with a vertical wall on a flat bottom (Fig. 1b) by applying a circulation theorem (see “Methods” section). However, their configuration is not directly applicable to reality, as the MIC is composed of a chain of islands over a sloping submarine ridge rather than a single island. Further, it is not even evident whether a streamfunction contour can be identified along the EKC that hits the MIC and forms a bifurcation point. For example, a recent simulation of the Ocean General Circulation Model for the Earth Simulator (OFES) with a 1/30° grid resolution^12, 13^ demonstrated a reversed + 1.1 Sv Pacific-ward transport through the northern strait, coupled with a − 0.8 Sv Okhotsk-ward current via the southern strait (Supplementary Fig. S1). By observing the streamfunction, we found that the EKC overshoots bypassing the two deepest straits without forming a bifurcation on the MIC (Supplementary Fig. S1), resulting in spurious results. This was apparently a hidden problem that was uncovered through the optimized physics of cutting-edge ocean general circulation model (OGCM) approaches, considering that the OFES with a coarser resolution (1/10°) reproduced exchange transport dynamics that were consistent with the observed estimates^7^ (Supplementary Fig. S1).

In this study, we demonstrate that tidal forcing is the missing mechanism that drives interbasin exchange caused by the partial intrusion of the EKC. By adopting an ocean model (CCSR Ocean Component model, hereinafter referred to as the COCO^14^; see “Methods” section and Matsuda et al.^15^) with a curvilinear coordinate system, a resolution of approximately 5 km (1/20°) around Kuril Islands, and accounting for diurnal tides in the simulation (referred to as the tidal case), the model estimated a throughflow transport of −4.0 Sv through the northern strait and + 8.7 Sv via the southern strait (Fig. 1c), thus realistically reproducing the observed estimates^7^. However, once the tidal forcing was turned off (referred to as the non-tidal case), the throughflow transport almost vanished, as illustrated in Fig. 1c, which represented a spurious result similar to the OFES at a 1/30° resolution mentioned above. This indicates that tidal forcing is an indispensable driver of interbasin exchange. The Kuril Islands region features vigorous tidal oscillations and currents^16^ dominated by the diurnal tides^9^, of which the tidal current’s amplitude sometimes exceeds 1.5 m s^−1^. In a previous study, by implementing a numerical model that only accounted for tides in which the EKC is absent, Nakamura et al.^9^ estimated the net exchange transport to be + 0.4 Sv and + 0.9 Sv through the northern and southern straits, respectively, both of which were Pacific-ward. That is, the tide-only model did not accurately represent the proper exchange transport dynamics either. This implies that the interaction between diurnal tides and the EKC is an essential driver of interbasin exchange. Here, we present a new mechanism for tidal control on the EKC’s pathway that leads to the partial intrusion to the Sea of Okhotsk from a potential vorticity (PV) dynamics standpoint.

## Results

### Overview of the tidal and non-tidal flow field

**Figure 2:**
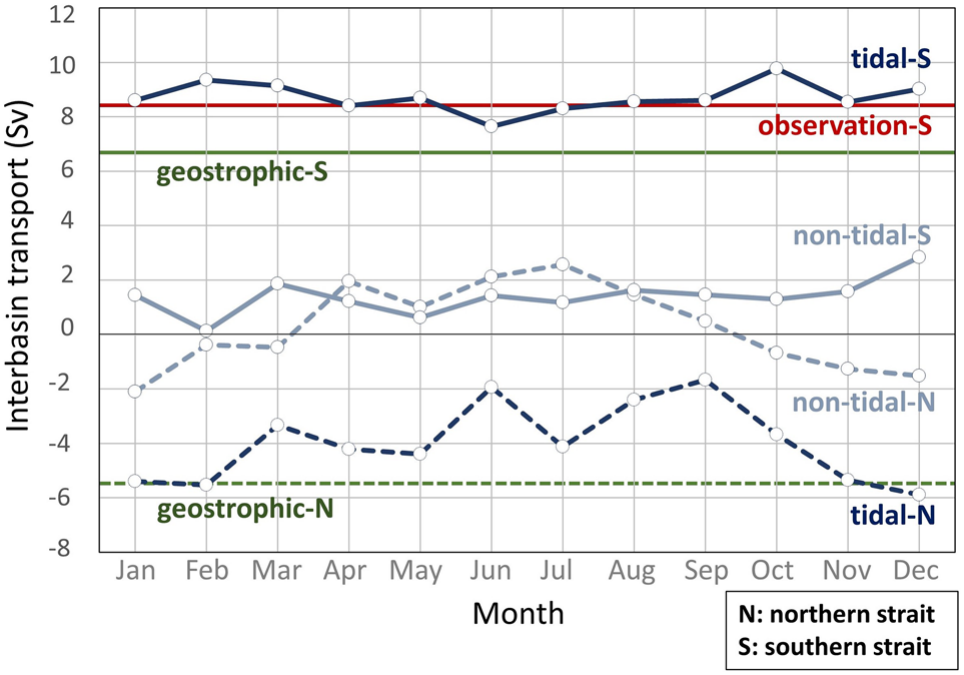
Transport through the northern and southern straits in the tidal and non-tidal cases. Seasonal volume transport through the northern strait (dashed-line, 153°E ~ 154°E) and the southern strait (solid-line, 150.4°E ~ 151.8°E) of the non-tidal case (grey-blue line) and the tidal case (navy-blue line). Positive values flow in a Pacific-ward direction. The red line denotes the observed transport of the southern strait^6^ in the summer and the green lines are the annually-averaged hydrographic transport value of the northern strait (dashed line) and southern strait (solid line)^7^. The abbreviations “N” and “S” correspond to the northern and the southern straits, respectively.

In this section, we analyzed the EKC path difference between the tidal and non-tidal cases and explored the roles of the tides on the exchange processes. The tidal case of the COCO, which was spun up by climatological forcing (see “Methods” section), estimates that the seasonal variation of the throughflow transport ranges from − 5.4 to − 1.9 Sv through the northern strait, and from + 7.6 to + 9.8 Sv via the southern strait (Fig. 2), which is consistent with the observed estimates^7^. Given that the exchange transport difference between the two cases is sufficient in both straits regardless of the season, we will focus on the generality of this difference induced by tides rather than seasonality. The June flow field was used in the following analysis, when the local wind stress curl around the MIC is weak (Supplementary Fig. S2) and the flow pattern is most typical compared to other months, where the EKC’s streamfunction contour encompasses most of the MIC, as illustrated in Fig. 1b (see Supplementary Figs. S3–S4 and circulation theorem in the “Methods” section).

**Figure 3:**
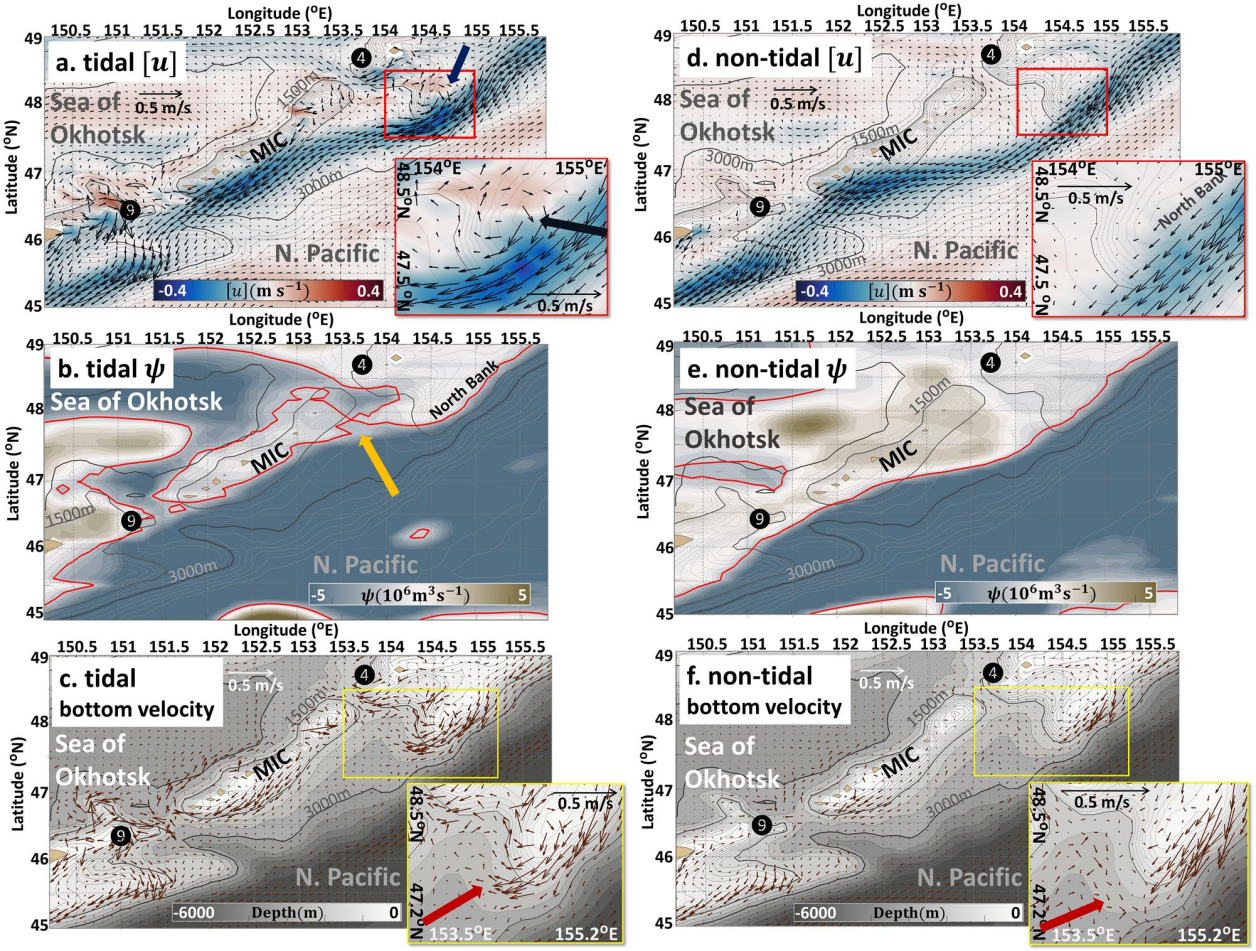
Non-tidal and tidal cases in June. Monthly-mean depth-averaged velocity [***u***], the streamfunction, and the bottom velocity of the bottom friction layer of the tidal and the non-tidal cases in June. (**a**) Flow pattern [***u***] of the tidal case. The grey contours denote the topography, where the contour interval is 150 m till 1500 m and continuously with 500 m intervals till 6000 m. The thick black contours denote the 1500 m and 3000 m depth, respectively. The circled numbers 4 and 9 indicate the northern (Kruzensterna) and southern (Bussol’) straits, respectively. The abbreviation MIC stands for Middle Island Chain. The topographic contour and circled numbers described herein will be used in the following figures of this study. The shade denotes the velocity of the zonal component of [***u***], in which red shade represents eastward and blue shade represents westward flow. The blue arrows indicate the tidally-rectified circulation. (**b**) The streamfunction ψ of the tidal case. The red contour is the streamfunction contour where $\psi =-1.5\times {10}^{6}$ m^3^ s^−1^. The streamfunction contour here represents ${\psi }_{1}$ in Fig. 1b. The yellow arrow indicates the bifurcation point. (**c**) Bottom velocity of the tidal case. The shade represents the bathymetry. (**d**) Same as (**a**) but for the non-tidal case. (**e**) Same as (**b**) but for the non-tidal case. (**f**) Same as (**c**) but for the non-tidal case. Note that the arrow scale of small window in (**f**) is slightly larger than that in (**c**) for vector clearness. The red arrows indicate the area in which the bottom velocity is reversed.

The depth-averaged velocity $\left[{\varvec{u}}\right]$ of the tidal case in June indicates an Okhotsk-ward flow through the northern strait and a Pacific-ward flow via the southern strait (Fig. 3a). Here, we define $[\blacksquare]=\frac{1}{H}{\int }_{-h}^{\eta }\blacksquare dz$ as a depth-averaged value, where $\blacksquare$ represents arbitrary variables, $z$ denotes the vertical coordinate, and $H=\eta +h$ represents the local water thickness, which equals the sum of the bottom depth *h* and the sea surface height $\eta$. The EKC deflects westward immediately after it passes the North Bank and approaches the MIC. Figure 3b depicts the flow field by a streamfunction $\psi$, defined by vertically-integrated velocity:

1 $$\frac{\partial \psi }{\partial x}=H\left[v\right], -\frac{\partial \psi }{\partial y}=H\left[u\right],$$

where $u$ and $v$ denote the zonal and meridional velocity, and $x$ and $y$ are the zonal and meridional coordinates, respectively. The streamfunction contour $\psi =-1.5\times {10}^{6}$ m^3^ s^−1^ extends from the North Bank toward the northern strait along with the EKC (red contour in Fig. 3b). At the same time, $\psi =-1.5\times {10}^{6}$ m^3^ s^−1^ surrounds the MIC. The two contours encounter approximately at 47.8°N, 153.6°E (indicated by a yellow arrow in Fig. 3b), forming a bifurcation point. This indicates that the transport of the EKC represented by $-1.5\times {10}^{6}<\psi \lesssim 0$ m^3^ s^−1^ is forced to flow through the northern strait. The geometrical features of the streamfunction in the tidal case agrees with those of Fig. 1b, in which the partial intrusion of the EKC occurs.

In contrast, the EKC in the non-tidal case bypasses most of the MIC (Fig. 3d,e). As a result, interbasin exchange is almost abolished, with weak Pacific-ward throughflows via both the northern and southern straits (Fig. 3d). The streamfunction depicts a clockwise flow pattern encompassing the MIC that blocks the interbasin exchange, and no bifurcation point forms in this case (Fig. 3e). We observed that the 1/30° resolution OFES (which does not incorporate tidal forcing) also displays a similar clockwise circulation surrounding the MIC, which makes the EKC bypass the deep straits (Supplementary Fig. S1) in a manner resembling the non-tidal case. These non-tidal results markedly contrast with those of the tidal case, implying that tidal forcing plays an essential role in the partial intrusion of the EKC.

One of the main differences between the tidal and non-tidal cases is the presence of seamount trapped waves in the former that propagate in the clockwise direction around topographic features such as the MIC and the North Bank^9, 17^ (see Supplementary Fig. S5 and Supplementary Movie 1). Further, a tidal-period-mean velocity indicates that tidally-rectified, clockwise circulations are generated around the islands and seamounts^9^. The tidally rectified circulation is prominent over the North Bank of the tidal case (compare Fig. 3a, blue arrows point, and Fig. 3d), which has been evidenced in the study site by the trajectories of Argo floats and surface drifters^11^.

To discuss the deflection of the EKC by interaction with these tidal motions, we focused on the bottom velocity around the North Bank. By comparing Fig. 3c,f, we found that the bottom velocity is much stronger in the tidal case, whereas in the non-tidal case, the along-slope bottom velocity is very weak or even reversed downstream of the North Bank (Fig. 3f; indicated by red arrows). Therefore, the EKC is hardly constrained by the topography in the downstream region in the non-tidal case, and thus the EKC overshoots passing by the deep straits at both ends of the MIC. In other words, bottom steering occurs due to tides, which forces the EKC to bend toward the northern strait after passing the North Bank, leading to the intrusion of the EKC water into the Sea of Okhotsk. The question now is why and how such a bottom steering of the EKC occurs in the tidal case. Is the bottom steering of the EKC a result of the trapped waves and/or the tidally-rectified circulation?

### Transition experiment from non-tidal to tidal state

To clarify the mechanisms of tidally-induced interbasin exchange, a transition experiment from the non-tidal state to the tidal state was conducted. Particularly, we focused on the processes that lead to the bottom steering of the EKC around the North Bank. The conditions on the last day of June of the non-tidal case were taken as the initial conditions for the transition experiment. Then, the tidal forcing was activated, and the model was executed for another 65 days (see the “Methods” section). Wind and heat fluxes were kept constant as those of June during the experiment. By doing this, the transition experiment would reveal the mechanisms by which tidal forcing produces the EKC’s bottom steering and shifts its pathway.

**Figure 4:**
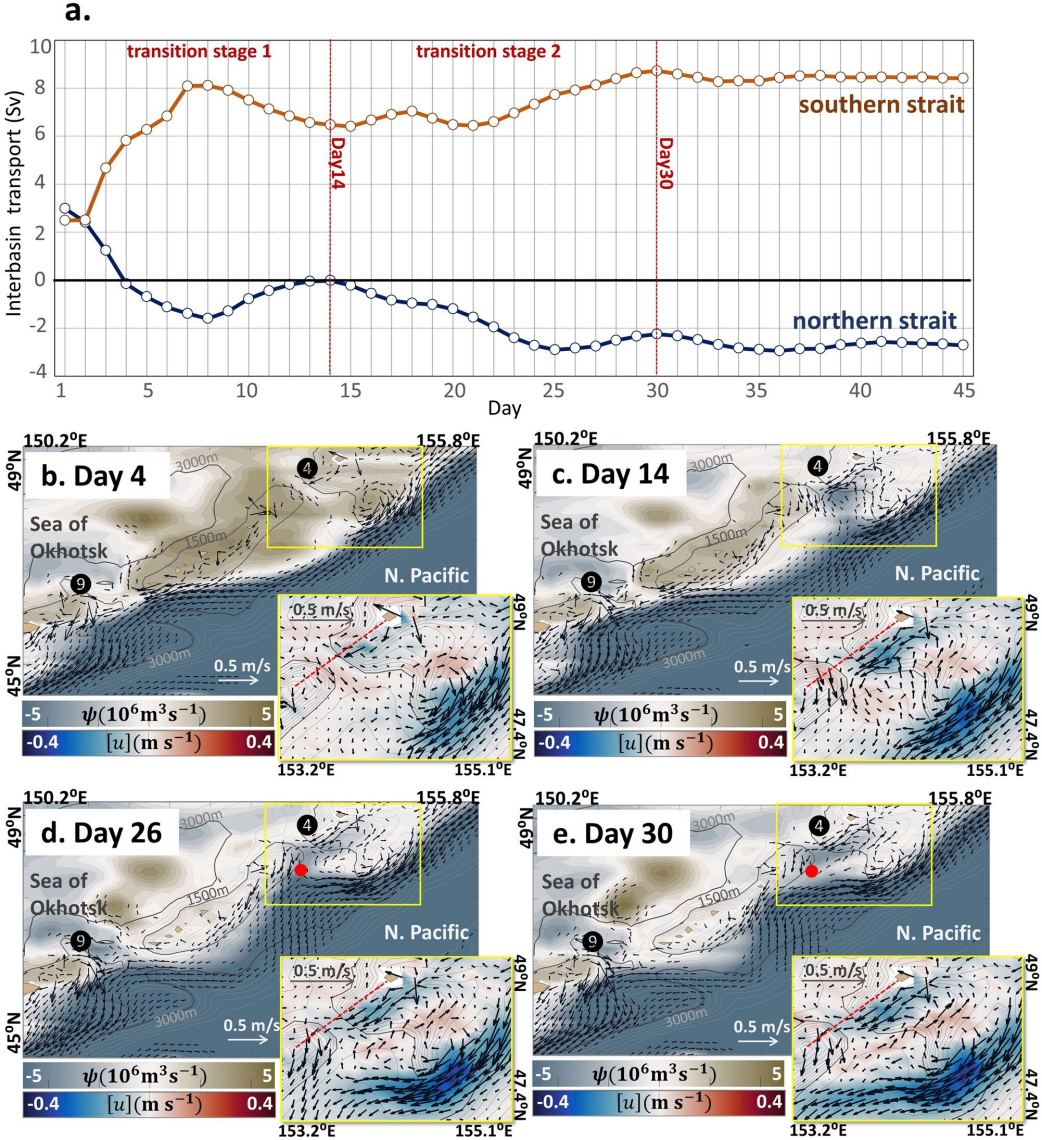
Exchange transport and evolution of the EKC during the transition experiment. (**a**) Evolution of volume transport through the northern strait (blue line) and the southern strait (orange line) from Day 1 to Day 45 during the experimental period. Positive values indicate Pacific-ward flows and negative values are Okhotsk-ward. The transition stage 1 is the period between Day 1 and Day 14, followed by stage 2 before Day 30. (**b**–**e**) The flow pattern during the transition experiment. (**b**) Day 4 state, which illustrates a bi-directional transport at the northern and southern straits. Vectors denote the depth-averaged velocity field [***u***], and only the one with speed ($\sqrt{{\left[u\right]}^{2}+{\left[v\right]}^{2}}$) over 0.05 m s^−1^ is shown. The shade denotes the streamfunction $\psi$. Tidally-rectified flow is also observed over the North Bank. The small panel represents an enlarged view of the [***u***] field as vectors around the northern strait and the North Bank with the shade as zonal velocity, all vectors are shown. The red dashed line denotes the section where transport was estimated in this study. The grey contours denote the topography, where the contour interval is 150 m till 1500 m and continuously with 500 m intervals till 6000 m. The thick black contours denote the 1500 m and 3000 m depth, respectively. (**c**) Same as (**b**) but for Day 14, (**d**) Day 26, and (**e**) Day 30. The shifting of the EKC pathway is illustrated in the panels (**d**) and (**e**). The red dots in (**d**) and (**e**) denote the bifurcation points.

Transport through the two straits is both Pacific-ward initially with approximately + 3 Sv according to the June state of the non-tidal case. After accounting for tidal forcing on Day 0, the transport through the northern and southern straits varied until Day 30 and reached an almost steady state thereafter (Fig. 4a). We may divide the period of the transition into two stages on Day 14. The period between Day 0 and Day 14 represents an initial impact by imposing the tidal forcing. In the northern strait, the Okhotsk-ward inflow (with a negative sign) increases up to Day 8 but decreases afterward until Day 14, when the transport via the northern strait exhibits 0 Sv. From Day 14 to Day 26, transport through the northern strait increases again and finally achieves a steady-state with − 2.8 Sv. The transport through the southern strait behaves similarly, displaying a two-stage development. These periods will hereinafter be referred to as “transition stage 1” and “transition stage 2”, respectively.

Transition stage 1 is characterized by the generation of seamount trapped waves (Supplementary Movie 1) and tidally rectified circulation around islands and seamounts (Fig. 4b–e and Supplementary Movie 2). For example, a bi-directional flow occurs in both the northern and southern straits (Fig. 4b). However, $\psi$ displays a large positive value encompassing the MIC which still forces the EKC to bypass these straits. This suggests that during stage 1, the tidal rectification due to seamount trapped waves is the main factor that drives the development of transport through straits, as discussed in Nakamura et al.^9^ (see Supplementary Movie 2). In the upstream surrounding the North Bank, an anticyclonic circulation due to tidal rectification forms on Day 4. However, it does not yet alter the EKC path.

During transition stage 2, the EKC starts bending on Day 14 immediately downstream of the North Bank (Fig. 4c and Supplementary Movie 2). As the EKC bends and collides with the northern part of the MIC, the Okhotsk-ward throughflow becomes predominant in the northern strait. By Day 26, the EKC approaches the MIC steered by relatively shallow bathymetric contours (Fig. 4d). The EKC then bifurcates (indicated by the red dots in Fig. 4d–e) when it encounters approximately the 1500 m isobath surrounding the MIC, where the value of $\psi$ decreases significantly compared with the initial value. Because of the formation of the bifurcation point, the Okhotsk-ward throughflow occurs via the northern strait similarly to that illustrated in Fig. 1b. Interbasin transport tends to become steady after Day 26. The flow pattern of the Okhotsk-Pacific exchange on Day 65 is almost the same as that of the tidal case. This experiment demonstrates that the interaction between tidally-induced circulation and the EKC over the North Bank drives the interbasin exchange in the tidal case.

### Tidal-period-mean PV budget of the EKC over the North Bank

To investigate the dynamic effects of tides on the EKC pathway, we diagnosed the EKC via the barotropic PV:

2 $$q=\frac{f+[\zeta ]}{H},$$

where $f$ is the Coriolis parameter, and $\left[{\upzeta }\right]={\varvec{k}}\cdot \nabla \times \left[{{\varvec{u}}}\right]$ is the depth-averaged relative vorticity where ${\varvec{k}}\,{\mathrm{and}}\, \nabla$ denote unit vectors in the vertical direction and the horizontal gradient operator $\nabla =(\partial /\partial x,\partial /\partial y)$, respectively. A tidal period mean of the PV equation yields the following Eq. ^18^:

3 $$\underbrace {{\frac{{\partial \bar{q}}}{{\partial t}}}}_{{tendency\,of\,PV}} + \underbrace {{\overline{{[\user2{u}]}} \cdot \nabla \bar{q}}}_{{tidal\,period\,mean\,PV\,advection\,(mean\,PV\,advection)}} = \underbrace {{ - \frac{{\overline{{J(\chi ,H^{{ - 1}} )}} }}{H}}}_{{JEBAR}} + \underbrace {{\frac{{\nabla \times \frac{\user2{\tau }}{{\rho _{0} H}}}}{H}}}_{{wind\,stress\,curl}} - \underbrace {{\frac{{\overline{{\nabla \times \frac{{C_{d} |\user2{u}|\user2{u}}}{{\rho _{0} H}}}} }}{H}}}_{{bottom\,stress\,curl}} - \underbrace {{\overline{{[\user2{u}]^{t} \cdot \nabla q^{t} }} }}_{{tidal - rectification}} ,$$

where ${\varvec{\tau}}$ is wind stress, ${\rho }_{0}$ is the referential density, ${C}_{d}$ is the bottom drag coefficient, and $\chi$ is defined by:

$$\chi =\frac{g}{{\rho }_{0}}{\int }_{-h}^{\eta }\rho z dz,$$

where $\rho$ is density and $g$ is gravity acceleration. $\overline{\blacksquare}$ and ${\blacksquare}^{t}$ represent the mean and tidal component as a function of the diurnal tide period, and $J$ denotes the Jacobian of a given variable. The first term on the right-hand-side of Eq. (3) is the joint-effect-of-baroclinicity-and-relief (JEBAR). This represents the effects of baroclinicity on bottom pressure across the topography, which causes torque on the water column^18, 19^. Further, in Eq. (3), the original PV advection term $\overline{\left[{\varvec{u}}\right]\cdot \nabla q}$ is decomposed as follows:

4 $$\overline{\left[{\varvec{u}}\right]\cdot \nabla q}\approx \overline{\left[{\varvec{u}}\right]}\cdot \nabla \overline{q }+\overline{{\left[{\varvec{u}}\right]}^{t}\cdot \nabla {q}^{t}},$$

where $-\overline{{\left[{\varvec{u}}\right]}^{t}\cdot \nabla {q}^{t}}$ on the right-hand-side of Eq. (3) represents PV production due to tidal rectification. Given that the wind stress in the transition experiment is constant as a function of time, the tidal effects on the EKC’s PV budget can be evaluated by the three other terms in the right-hand-side of Eq. (3).

**Figure 5:**
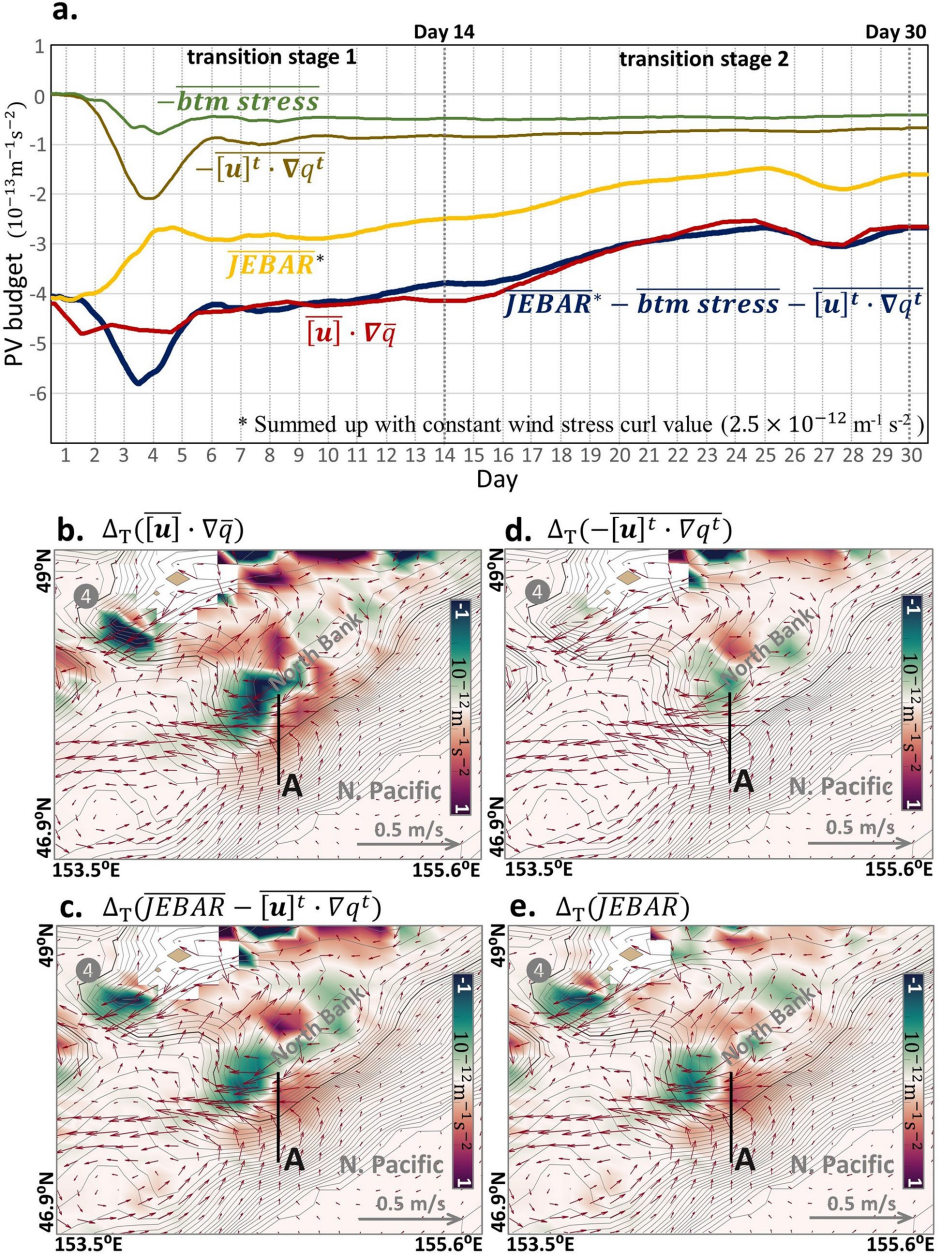
Evolution of PV budgets during the transition experiment. (**a**) Each term in the PV Eq. (3) from Day 1 to Day 30 in Section A in the transition experiment, where the EKC alters the pathway during the transition from the non-tidal case to the tidal case. The grass-green line, earth-yellow line, yellow line, and navy-blue line denote the bottom stress curl, tidal-rectification term, JEBAR term (including the constant wind stress curl), and the total of the right-hand side of the Eq. (3) including the constant wind stress curl, respectively. The red line represents the mean PV advection on the left-hand side of the Eq. (3). Vertical thick grey dashed lines indicate the date on Day 14 and Day 30, which are the beginning and end of transition period stage 2. (**b**–**e**) Distribution of terms in the PV equation based on the difference between Day 30 and Day 1 (Day 30–Day 1). The vectors also indicate the differences in depth-averaged velocity between Day 30 and Day 1. The black line denotes Section A. The grey contours denote the topography, where the contour interval is 150 m till 6000 m. The thick dark-grey contours denote the 1500 m depth. (**b**) Mean PV advection term. (**c**) Sum of JEBAR and tidal-rectification term. (**d**) Tidal-rectification term. (**e**) JEBAR term.

The tidal-period-mean PV advection $\overline{\left[{\varvec{u}}\right]}\cdot \nabla \overline{q }$ (hereinafter referred to as ‘mean PV advection’) balances well with the sum of the PV production terms (Fig. 5a) along Section A to the south of the North Bank, where the EKC starts deflecting westward. The mean PV advection $\overline{\left[{\varvec{u}}\right]}\cdot \nabla \overline{q }$ exhibits almost a constant value $\approx$−4.2 m$\times {10}^{-13}\mathrm{m}$^−1^ s^−2^ during transition stage 1, except for a negative peak between Day 2 and Day 6, when the difference with the sum of the PV production terms reaches a maximum. The difference corresponds to the local vorticity production $\partial \overline{q }/\partial t$, which represents the generation of clockwise, tidally-rectified circulation around the North Bank by $-\overline{{\left[{\varvec{u}}\right]}^{t}\cdot \nabla {q}^{t}}$ and exhibits a conspicuous peak during the same period.

In transition stage 2, the mean PV advection increases from − 4.2$\times {10}^{-13}$m^−1^ s^−2^ to − 2.6$\times {10}^{-13}\mathrm{m}$^−1^ s^−2^ when the EKC shifts. The tidal rectification and bottom friction terms remain almost constant in the PV budget during transition stage 2. The wind stress term is constant throughout the experiment despite having a high value (2.5$\times {10}^{-12}\mathrm{m}$^−1^ s^−2^). Therefore, the only term that changes with time is the JEBAR, which can balance with changes in mean PV advection.

Maps of the PV budget difference between Day 30 and Day 1, defined by ${(PV)}_{Day30}-{(PV)}_{Day1}$, are illustrated in Fig. 5b–e. Since $\overline{q}\approx {f }_{0}/H$, where ${f}_{0}$ is the Coriolis parameter around the North Bank, the difference of the mean PV advection in terms of time is approximated as follows:

5 $${\Delta }_{T}\left( \overline{\left[{\varvec{u}}\right]}\cdot \nabla \overline{q }\right)\approx {\Delta }_{T}\overline{\left[{\varvec{u}}\right]}\cdot \nabla \frac{{f}_{0}}{H},$$

where ${\Delta }_{T}$ is an operator representing the difference between Day 30 and Day 1. According to Eq. (5), the difference in the mean PV advection corresponds to the velocity difference between Day 30 and Day 1, ${\Delta }_{T}\overline{\left[{\varvec{u}}\right]}$ , across PV (or bathymetric) contours. Since ${\Delta }_{T}\left( \overline{\left[{\varvec{u}}\right]}\cdot \nabla \overline{q }\right)>0$ in Section A, ${\Delta }_{T}\overline{\left[{\varvec{u}}\right]}$ takes place in the upslope direction ($\nabla \frac{{f}_{0}}{H}>0)$, as seen in the vectors in Fig. 5b. That is, the positive difference in the mean PV advection around Section A corresponds to an upslope transition of the EKC pathway.

The difference of the mean PV advection around the North Bank balances well with the sum of the change of the tidal rectification and JEBAR terms in the right-hand-side of Eq. (3) (Fig. 5b,c). Particularly, the JEBAR’s contribution (Fig. 5e) is dominant around Section A, which can be expressed as follows:

6 $${\Delta }_{T}{\left(\overline{\left[{\varvec{u}}\right]}\right)}_{A}\cdot \nabla \frac{{f}_{0}}{H}\approx -{\left(\overline{\frac{J\left({\Delta }_{T}\chi , {H}^{-1}\right)}{H}}\right)}_{A},$$

where the subscript A denotes the region around Section A, and

7 $${\Delta }_{T}\chi =\frac{g}{{\rho }_{0}}{\int }_{-h}^{\eta }{\Delta }_{T}\rho z dz.$$

This result indicates that the JEBAR change due to ${\Delta }_{T}\chi$, associated with the density change, ${\Delta }_{T}\rho$, in a given water column drives the EKC’s upslope transition over the North Bank across PV contours. However, the source of the JEBAR change remained to be determined.

### Source of JEBAR change for the EKC’s path shift

The JEBAR term is incorporated into PV Eq. (3) as a wind stress curl that generates flows across PV contours once a density field is specified^18^. In the tidal case, JEBAR forces the EKC upslope to relocate and bend it toward the MIC, resulting in interbasin exchange. Here, we identified the impact of tidal forcing that alters the density field, leading to JEBAR changes over the North Bank.

**Figure 6:**
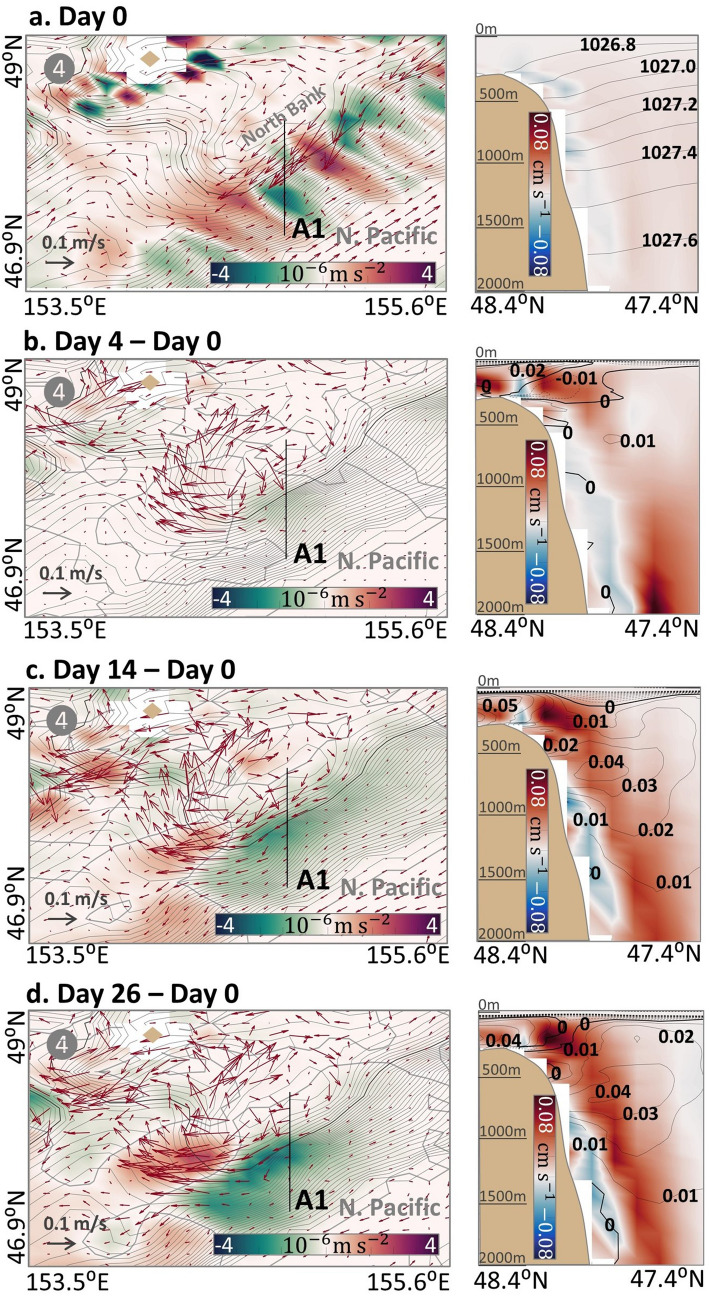
Pressure torque generation. Bottom pressure torque (left panel) and the formation of the overturning cell on the North Bank (right panel) during the transition experiment. (**a**) Day 0 state. Left panel: Bottom pressure torque (shade) and bottom velocity (vectors). Right panel: Vertical velocity (shade) and density (kg m^−3^, contours). (**b**) Difference between Day 4 and Day 0 (Day 4–Day 0). Left panel: bottom pressure torque differences (shade) and bottom velocity differences (vectors). The thick grey contour represents the division of positive and negative values of the bottom torque differences. Topography contours are as same as Fig. 5. Right panel: difference in vertical velocity (shade) and density (kg m^−3^, contours) in Section A1, shown as a black line in the left panel. Solid (dashed) contours denote the positive (negative) values of density differences from Day 0. (**c**) Same as (**b**) but for the difference between Day 14 and Day 0 (Day 14–Day 0). (**d**) Same as (**b**) but for the difference between Day 26 and Day 0 (Day 26–Day 0).

To examine the mechanism of JEBAR increase, we inspected the density structure in Section A1 (Fig. 6, right panels), which is 15 km upstream of Section A, and identified an overturning cell on the slope depicted by the vertical velocity. The formation of the overturning cell associated with the tidally-rectified circulation has been widely discussed in previous studies^20–22^. The overturning cell in Fig. 6 is composed of a downwelling on the slope of a seamount and upwelling around the outer rim of the tidally-rectified circulation. Since the overturning redistributes the density field vertically and horizontally, the JEBAR change due to ${\Delta }_{T}\chi$ should occur through the density change ${\Delta }_{T}\rho$ according to Eq. (7).

Further, we introduced the bottom pressure torque $J({p}_{b}/{\rho }_{0},H)$ to explore the effect of bottom steering on the EKC (Fig. 6, left panels), where ${p}_{b}$ denotes bottom pressure. ${p}_{b}$ is related to $\chi$ through ${p}_{b}=[p]-\frac{{\rho }_{0}\chi }{H}$, where $[p]$ represents depth-averaged pressure^18, 19^. The relationship between the bottom pressure torque and the overturning cell is illustrated in Fig. 6. The overturning cell is generated on the slope along Section A1 as soon as the tidally-rectified circulation forms by Day 4 (Fig. 6a,b). The bottom pressure torque exhibits a supply of negative vorticity at 1000–2000 m depths around A1, which likely turns the bottom velocity clockwise. This strongly indicates the cross-slope upwelling reinforces the bottom pressure torque by shoaling isopycnals up to ~ 700 m (Fig. 6c,d). Concurrently, an along-isobath bottom flow occurs downstream of Section A1 around the North Bank at depths of 1000–1500 m, where the positive bottom pressure torque (on Day 0) is canceled by the negative torque supply until the end of transition stage 2. The bottom steering drives the EKC in an upslope direction and reaches a similar state to that of the tidal case (Fig. 3a). Since the density change associated with the overturning cell, which is generated by the tidal rectification, produces the JEBAR’s change for the transition of the EKC, we refer to this process as a “tidally driven JEBAR mechanism.”

### Tidally-modified EKC drives the interbasin exchange

**Figure 7:**
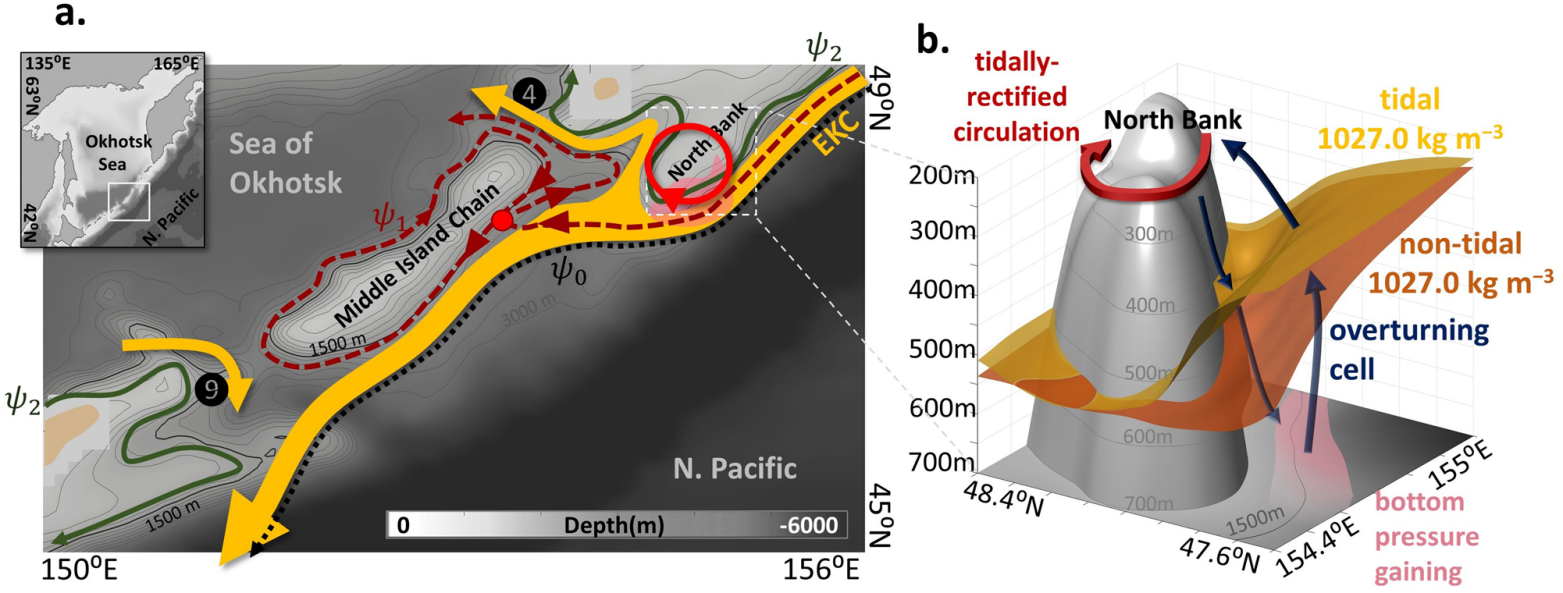
Schematic view of the Okhotsk-Pacific exchange system. (**a**) Sketch of the Okhotsk-Pacific exchange system incorporating tidal forcing. The underground area represents the topography focused on the MIC area, which is represented by the grey contours (250 m depth intervals reaching 3000 m). The dark-grey contour indicates the 1500 m depth. The dashed black line, dashed red line, and solid army-green line denote the streamfunction contour offshore boundary of the EKC (${\psi }_{0}$), surrounding the MIC (${\psi }_{1}$), and surrounding the islands northern/southern than the MIC (${\psi }_{2}$), respectively. The yellow arrow denotes the EKC pathway with the exchange transport in the tidal case, which follows the streamfunction contour ${\psi }_{1}$ as the core axes. The red dot on the MIC denotes the bifurcation point. The red arrowed circle above the North Bank represents the tidally-rectified circulation caused by the propagation of seamount trapped waves. The transparent pink area is the region where the PV is supplied by the JEBAR along with the formation of tidally-rectified circulation. (**b**) Generation of the overturning cell and the isopycnal elevation formed through the generation of the tidally-rectified circulation over the North Bank area. The yellow and dark-orange surfaces indicate the 1027.0 $\text{kg m}^{{ - 3}}$ isopycnal surfaces of the tidal case and the non-tidal case, respectively. The red circle represents the tidally-rectified circulation and the blue arrows denote the overturning cell which is caused by the tidally-rectified circulation.

Finally, Fig. 7 summarizes our overarching findings. In the absence of tidal forcing, the EKC tends to bypass the deep straits and does not induce interbasin exchange. Once the tidal forcing is incorporated, however, the EKC is forced to bend westward after passing the North Bank, and approaches the MIC along with the streamfunction contour ${\psi }_{1}$ as depicted in Fig. 7a. Afterward, the bifurcation point on ${\psi }_{1}$ occurs around the MIC and the EKC branches northward and southward (Fig. 7a). The northern branch of the EKC passes through the northern strait Okhotsk-ward and drives the interbasin transport. Upstream of the MIC, the westward deflection of the EKC occurs as a result of steering by the topography of the North Bank when the tidal forcing is imposed (Fig. 7b). The cross-slope overturning cell in response to the tidally-rectified circulation raises the bottom pressure torque via shoaling of the isopycnals at depth. Increases in the bottom pressure intensified the bottom velocity along isobaths and relocated the EKC pathway across the PV contours by the JEBAR term.

High-resolution simulations typically improve the representation of the flow through straits of WBCs, as described by Metzger and Hurlburt^23^. Concretely, the authors found that the existence of an underwater bank in the Luzon Strait blocks the intrusion of the Kuroshio as the model resolution increased, which is consistent with the observations. This phenomenon was defined as the “blocking effect”^23^ and resembles our non-tidal case. However, as discussed in this study, improving the model resolution does not necessarily reproduce the interbasin exchange accurately as shown in the non-tidal case because of the non-linearity. Sheremet^24^ evaluated whether a WBC leaps across a strait caused by the current’s inertia, overcoming the westward bending due to planetary Rossby waves. In linear models, planetary Rossby waves enforce the EKC deflecting to the MIC and drive the interbasin exchange^10^. In high-resolution models, however, inertia of WBCs tends to dominate over the Rossby wave dynamics and thus the interbasin exchange is blocked as shown in the non-tidal case. Clearly, a better understanding of the physical processes that drive the WBCs is necessary as the model resolution increases.

Our study proposes a novel mechanism for the interaction between tidally-rectified circulation and the boundary current through JEBAR. We also demonstrated that the interaction between boundary currents and tides, which is usually lacking in modeling studies, is key to improve high-resolution OGCM performance.

## Discussion

Our study elucidated the impact of tidal forcing on the relocation of the EKC pathway due to the changes in JEBAR, which consequently leads to the partial intrusion of the EKC into the Sea of Okhotsk. However, the diurnal tides not only generate rectified circulation but also cause strong vertical mixing along the Kuril Islands by breaking of internal tides^9, 17^. Although the impact of tidally-induced vertical mixing along the Kuril Islands on the North Pacific’s thermohaline circulation was suggested^25, 26^, its influence on the Okhotsk-Pacific exchange system was seldom mentioned.

Vertical mixing observed along the Kuril Islands is as high as 25 cm^2^ s^−1 ^^27^, which is two orders of magnitude stronger than that in the ocean int erior^27^. In the COCO, vertical mixing was parameterized as the vertical diffusivity coefficients evaluated by the turbulent closure scheme^28^. The vertical diffusivity coefficients around the islands were approximately 23.9 cm^2^ s^−1^ and 13.6 cm^2^ s^−1^ in the tidal and non-tidal cases, respectively (see Supplementary Fig. S6). To evaluate the impacts of vertical mixing with no tidal oscillations on the Okhotsk-Pacific exchange system, we conducted an experiment in which the vertical diffusivity coefficients extracted from the tidal case were substituted with those of the non-tidal case (Supplementary Fig. S6; see the “Methods” section). This case is henceforth referred to as the “non-tidal-TM case,” where TM denotes Tidal Mixing.

**Figure 8:**
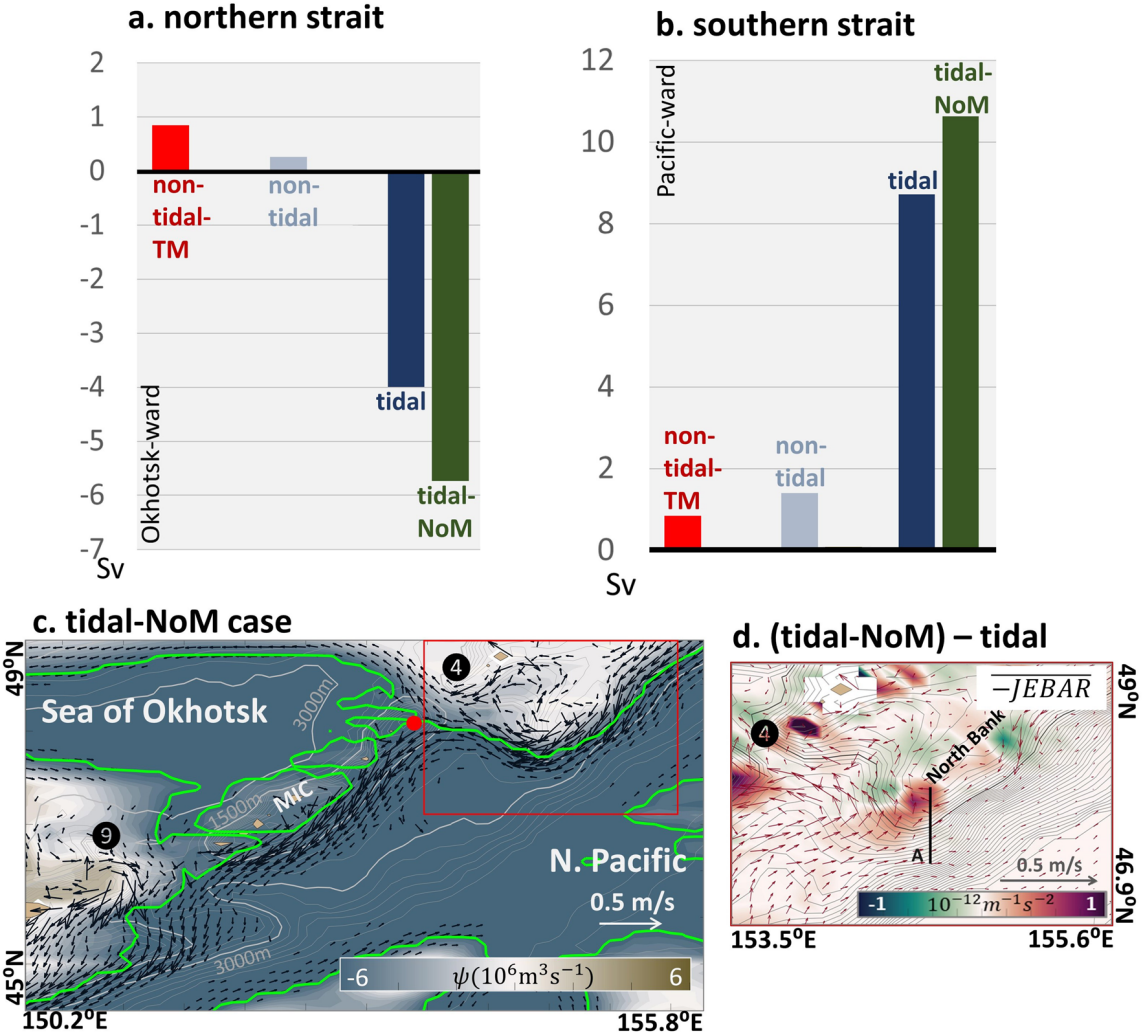
TM case and NoM case experiments. (**a**,**b**) Annual volume transport rate of the non-tidal-TM, the non-tidal, the tidal, and the tidal-NoM cases. (**a**) and (**b**) Volume transport via the northern and the southern straits, respectively. Positive values indicate Pacific-ward transport. Red, grey-blue, navy-blue, and army-green bars denote the non-tidal-TM, the non-tidal, the tidal, and the tidal-NoM cases, respectively. (**c**) Plan view of the depth-averaged velocity (vector), in which only the one with speed ($\sqrt{{\left[u\right]}^{2}+{\left[v\right]}^{2}}$) over 0.05 m s^−1^ is shown, and the streamfunction (shade) of the tidal-NoM case. The green contour denotes the streamfunction contour $\psi =-5.81\times {10}^{6}$ m^3^ s^−1^. The red dot represents the bifurcation point. Topography contours are as same as Fig. 3 but note that the scale of shading is different from Figs. 3 and 4. (**d**) Differences in JEBAR (shade) and depth-averaged velocity (vector) between the tidal-NoM and the tidal cases. The black line denotes Section A. The JEBAR is higher in the tidal-NoM case, which corresponds with the EKC pathway adjustment. The EKC shifts to the relatively shallower contour of the location where the positive value occurred.

We found that the interbasin exchange in the non-tidal-TM case exhibited the most unrealistic throughflow transport estimates of + 0.9 Sv and + 0.8 Sv at the northern and southern straits, respectively (Fig. 8a,b). This scenario exhibited an intense clockwise circulation surrounding the MIC (Supplementary Fig. S7), which was similar to the results of the non-tidal case (Fig. 3e). Therefore, enhanced vertical mixing does not drive the intrusion of the EKC into the Sea of Okhotsk. That is, vertical mixing is not a factor that causes interbasin exchange in the tidal case.

For further sensitivity testing, an experiment in which the vertical diffusivity coefficient was set to zero was conducted with the tidal-case configuration (henceforth referred to as the “tidal-NoM” case, where “NoM” means no-mixing; see the “Methods” section). The interbasin exchange in the tidal-NoM case was the largest among all cases (Fig. 8a,b). The EKC exhibits a sharp deflection around the North Bank and bifurcates at the MIC’s northern corner by following the streamfunction contour $\psi =-5.81\times {10}^{6}$ m^3^ s^−1^, resulting in a strong Okhotsk-ward throughflow transport (Fig. 8c).

According to the tidally driven JEBAR mechanism, the northward relocation of the EKC toward the MIC is expressed by the PV supply via JEBAR on the North Bank’s eastern slope. The tidal-NoM case shows a larger JEBAR value around Section A than that of the tidal case (Fig. 8d). The vertical profiles of density and along-slope velocity on Section A indicate that the EKC in the tidal-NoM case locates further upslope compared with that of the tidal case (Supplementary Fig. S6). The effects of the tidally-rectified circulation on the EKC are enhanced in the tidal-NoM case compared with the tidal case by strengthening the tidally driven JEBAR mechanism. Considering the results of the sensitivity experiments, we concluded that tidally-induced vertical mixing tends to reduce the interbasin transport between the Sea of Okhotsk and the North Pacific.

Vertical mixing is often incorporated in OGCMs through parameterizations over rough topography to estimate the global distribution of tidal energy available for turbulent mixing^29, 30^. In contrast, the dynamic effects of tides such as the interaction between western boundary currents and tidal motions are often overlooked in basin-wide or global-scale modeling. However, as discussed in the introduction, high-resolution OGCMs are uncovering inconspicuous problems inherent to relatively low-resolution models due to improvements in physics modeling.

Our results suggest that the tidal forcing and the resulting tidally driven JEBAR mechanism are a missing piece for high-resolution OGCMs in which strong boundary currents and topographic features are accurately characterized in high-latitude areas. The tidally driven JEBAR mechanism may also apply to various regions where both boundary currents and intensive diurnal tides coexist. For example, the Aleutian Arc and the Antarctic continental shelves are candidates; in the former, the Alaskan Stream controls the interbasin exchange between the Bering Sea and the North Pacific^31, 32^, whereas in the latter case, the Antarctic Slope Current regulates the heat transport to the ice shelf^33, 34^.

## Methods

### Circulation theorem

The vertical averaged the momentum equation as a function of depth $z$ from $z=-H$ to $z=0$, where $-H$ is a depth of bottom, can be expressed as follows:

$$\frac{\partial }{\partial t}{\int }_{-H}^{0}{\varvec{u}}dz+{\int }_{-H}^{0}\left(f+\zeta \right)\left({\varvec{k}}\times {\varvec{u}}\right)dz=-{\int }_{-H}^{0}\nabla \left(\frac{p}{{\rho }_{0}}+\frac{{|{\varvec{u}}|}^{2}}{2}\right)dz+\frac{{{\varvec{\tau}}}_{{\varvec{s}}}}{{\rho }_{0}}-\frac{{{\varvec{\tau}}}_{{\varvec{b}}}}{{\rho }_{0}}+{\int }_{-H}^{0}\mathcal{F}dz,$$

where ${\varvec{u}}$ denotes a horizontal velocity vector, $\nabla$ is the horizontal gradient operator, $f \mathrm{and }\zeta$ denote the planetary and relative vorticity, respectively, ${\rho }_{0}$ is a typical density, and $p$ represents pressure. ${{\varvec{\tau}}}_{{\varvec{s}}}$ and ${{\varvec{\tau}}}_{{\varvec{b}}}$ denote wind stress and bottom stress, respectively, and $\mathcal{F}$ denotes horizontal viscosity. Afterward, taking a circulation integral along a closed constant-depth contour $H={H}_{0}$, circulation can be expressed as follows:

$$\frac{\partial }{\partial t}\oint {\int }_{-{H}_{0}}^{0}{\varvec{u}}\cdot {\varvec{t}}dzdl+\oint {\int }_{-{H}_{0}}^{0}\left(f+\zeta \right){\varvec{u}}\cdot {\varvec{n}}dzdl =\oint \frac{{{\varvec{\tau}}}_{{\varvec{s}}}}{{\rho }_{0}}\cdot {\varvec{t}}dl-\oint \frac{{{\varvec{\tau}}}_{{\varvec{b}}}}{{\rho }_{0}}\cdot {\varvec{t}}dl+\oint {\int }_{-{{\varvec{H}}}_{0}}^{0}\mathcal{F}\cdot {\varvec{t}}dzdl,$$

where $l$ is an along-isobath coordinate, and ${\varvec{t}}$ and ${\varvec{n}}$ denote unit vectors along- and cross-isobath coordinates, respectively. Kida and Qiu^10^ derived a streamfunction value on the wall of a single island over a flat bottom by evaluating

$$\oint {\int }_{-{H}_{0}}^{0}\mathcal{F}\cdot {\varvec{t}}dzdl=0,$$

assuming a steady-state with spatially constant wind stress ${{\varvec{\tau}}}_{{\varvec{s}}}$ in a deep ocean where bottom stress ${{\varvec{\tau}}}_{{\varvec{b}}}$ is negligible. Note that the vorticity flux term (i.e., the second term in the left-hand-side) is identically zero along the island wall in their simple model.

In reality, the vorticity flux term across isobaths (e.g., 1500 m) is not zero, as the MIC is not a single island on a flat bottom but a chain of islands over a submarine ridge. Vorticity flux convergence may therefore occur and drive an anti-cyclonic circulation encompassing the MIC that blocks the interbasin exchange. This may potentially explain the spurious results of the recent high-resolution GCMs.

### CCSR ocean component model (COCO)

The CCSR Ocean Component Model (so-called COCO) is an ocean general circulation model developed by the Atmosphere and Ocean Research Institute of the University of Tokyo^14^. The present model configurations are the same as those of Matsuda et al.^15^, which covers the Pacific Ocean from 10°S to 67°N meridionally and 100°E to 90°W zonally. This includes all important marginal seas of the North Pacific, including the Sea of Okhotsk, the Bering Sea, the South China Sea, the Japan Sea, and the Indonesian Straits. The Sea of Okhotsk is configured to be high resolution (finer than 3 km in the northern shelf region and 5 km around the Kuril Islands that divide the Sea of Okhotsk from the North Pacific) by using a curvilinear coordinate grid. Vertical grids are totally 84 layers composed of 7 sigma coordinate layers shallower than 35 m, followed by 36 z-coordinates layers with 10 m thickness intervals, and 41 z-coordinate layers by thickness interval exponentially increases from 12 to 600 m. The model’s bottom topography is adapted from the Japan Oceanographic Data Center (JODC) and modified by Ono et al.^35^ and Matsuda et al.^15^ However, we eliminated the extra vertical diffusivity around the Kuril Islands added by Matsuda et al.^15^.

The atmospheric forcing that drives ocean and sea ice dynamics in the COCO model are the monthly-averaged climatology forcing from the Ocean Model Intercomparison Project (OMIP)^36, 37^ based on 15 years of daily reanalysis data from 1979 to 1993 obtained from the European Centre for Medium-Range Weather Forecasts^36^, coupled with monthly-averaged river runoff data from Dai and Trenberth^38^. The sea ice motion dynamics are based on elastic-viscous-plastic rheology. Readers may refer to Matsuda et al.^15^ for further details of the model configuration.

The tidal forcing of the K1 constituent is given by the tidal potential $\xi$ in the barotropic momentum equations such that

8 $$\frac{\partial {{\varvec{u}}}_{{\varvec{T}}}}{\partial t}+\left({{\varvec{u}}}_{{\varvec{T}}}\cdot \nabla \right){{\varvec{u}}}_{{\varvec{T}}}+f{\varvec{k}}\times {{\varvec{u}}}_{{\varvec{T}}}=-g\nabla \left({\alpha }_{0}\eta -{\beta }_{0}\xi \right)+{A}_{H}{\nabla }^{2}{{\varvec{u}}}_{{\varvec{T}}},$$

where ${{\varvec{u}}}_{{\varvec{T}}}$ is the barotropic velocity and ${A}_{H}$ is the horizontal eddy diffusivity. The tidal potential is written as $\xi =K{\text{sin}}2\phi \mathrm{cos}\left(\sigma t+\lambda \right)$ for the diurnal tide, where $\phi$ and $\lambda$ are the latitude and longitude, respectively, with the amplitude $K$= 0.14 m, frequency $\sigma =0.72921\times {10}^{-4}$ s^−1^, and ${(\alpha }_{0},{\beta }_{0})=\left(0.90, 0.69\right)$ ^39^. The model configuration for the non-tidal cases does not consider $\xi$ in the barotropic momentum equation. Please see Matsuda et al.^15^ for more details on the validation of simulated tidal currents.

In the first year of the model integration, the temperature and salinity were restored to the World Ocean Atlas 2009 (WOA09)^15^. We then span it up for 60 years by the climatology OMIP dataset same as Matsuda et al.^15^ but without tidal forcing. We used the model’s final outputs as our initial conditions. We next span it up for 45 years by the same climatology OMIP forcing with the tidal and non-tidal configurations according to Matsuda et al.^15^ as the tidal and non-tidal cases, respectively. The last year's model output was used for analysis.

### Ocean general circulation model for the earth simulator (OFES)

The ocean model output from the Ocean General Circulation Model for the Earth Simulator (OFES) 1/30°^12, 13^ was also used for this study. The model domain ranges from 100°E to 70°W and from 20°S to 68°N with 100 vertical levels, encompassing the marginal seas in the North Pacific. The bathymetry data was obtained by merging the General bathymetric Chart of the Oceans (GEBCO) (1 min)^40^ and JTOPO30^41^. Vertical mixing in the mixed layer was parameterized as the vertical diffusivity coefficients by Noh and Kim^28^. First, we conducted the climatological integration of the OFES 1/10° simulation for 15 years, which was 30 years spun up by the temperature and salinity data of WOA13 as the initial state^12^. Afterward, a hindcast OFES 1/10° simulation was conducted from 1979 until 2000. Ultimately, the OFES 1/30° simulation spanned from January 1, 2000, to December 31, 2003, which was forced by the historical atmospheric reanalysis data of the JRA-25^42^. Data corresponding to the year 2003 was used in this study.

### Transition experiment

The transition experiment was conducted by activating tidal forcing ($\xi$) in the momentum Eq. (8) for 65 days of simulation. The initial state of the experiment is the final day of June of the non-tidal case, and the rest of the forcing parameters, including heat flux, wind stress, and freshwater flux, remained the same as those in June. The impacts of tidal forcing with no influence from other forcing parameters were modeled by maintaining wind and heat forcing constant for 65 days from the end of June. The analyses were conducted using the model output recorded every 1.1975 (= 23.95/20) hours, where 23.95 h represents the K1-tidal-period.

### Vertical mixing experiments

The vertical mixing in the COCO model is modeled by a turbulent closure scheme by Noh and Kim^28^ such that

$${A}_{v}=Sql, \mathrm{and,} {K}_{v}=\frac{Sql}{{P}_{r}},$$

where ${A}_{v}$ is the vertical viscosity, and ${K}_{v}$ is the vertical diffusivity. $l$ and $q$ are turbulence length scale and root-mean-square velocity of turbulence, respectively. $S$ is a coefficient parameterized as $S= {S}_{0}/\sqrt{1+\alpha {R}_{it}}$, where ${S}_{0}$ is given as 0.39, $\alpha =3$ is a constant for proportionality, and ${R}_{it}$ is the turbulent Richardson number, ${R}_{it}={\left(Nl/q\right)}^{2}$, where ${N}^{2}$ is the buoyancy frequency, $-g{{\rho }_{0}}^{-1}\partial \rho /\partial z$. ${P}_{r}$ is the Prandtl number, where ${P}_{r}={P}_{r0}+\beta {R}_{i}$, ${P}_{r0}$ and $\beta$ are constants equal to 0.8 and 7, respectively, and ${R}_{i}$ is the Richardson Number, ${R}_{i}={N}^{2}/\left[{\left(\partial u/\partial z\right)}^{2}+{\left(\partial v/\partial z\right)}^{2}\right]$.

Here we designed two different types of model experiments, testing the vertical diffusivity function in water exchange transport. The first experiment was the non-tidal-TM case, which was executed by substituting the tidal case’s vertical diffusivity value into the non-tidal case monthly for 5 years to detect the influences of tidal mixing without residual tidal currents. Second, we conducted sensitivity tests enforcing the vertical diffusivity value to be zero in the tidal model configuration, which we referred to as the tidal-NoM case. The models were spun up for 5 years under climatological forcing. The model outputs of the last year were used for analysis in both settings.

## Supplementary Information

Supplementary Video 1.

Supplementary Video 2.

Supplementary Figures.

## Data Availability

Figures 1a, 3, 4b–e, 5b–e, 6, 7, and 8c–d were produced using Matlab (Matlab R2019b, https://www.mathworks.com/products/new_products/release2019b.html). The topography data is obtained from the COCO model (see the "Methods" section). Figure 1b is illustrated by PowerPoint (PowerPoint 2016, https://www.microsoft.com/ja-jp/download/details.aspx?id=53373). All data are available from the authors on reasonable request.
