# Supplementary material for: Tidally modified western boundary current drives interbasin exchange between the Sea of Okhotsk and the North Pacific
Source: Sci Rep. 2021 Jun 8;11:12037. doi: 10.1038/s41598-021-91412-y (PMC8187450; doi:10.1038/s41598-021-91412-y)
Supplement: Supplementary file 3 — Supplementary Figures. [file 41598_2021_91412_MOESM3_ESM.pdf]

Tidally modified western boundary current drives interbasin exchange between the Sea of Okhotsk and the North Pacific

Hung-Wei Shu, Humio Mitsudera, Kaihe Yamazaki,
Tomohiro Nakamura, Takao Kawasaki, Takuya Nakanowatari,
Hatsumi Nishikawa, Hideharu Sasaki

Supplementary Figures

Contents

Supplementary Figure S1. **Results of the OFES 1/30° model.** 2

Supplementary Figure S2. **Local wind-stress-curl line integrated along a 1500 m contour surrounding the MIC.** 4

Supplementary Figure S3. Streamfunction of the tidal cases. 5

Supplementary Figure S4. Streamfunction of the non-tidal cases. 7

Supplementary Figure S5. Propagation of seamount trapped waves. 9

Supplementary Figure S6. **Bottom velocity and vertical diffusivity distribution in each case.** 10

Supplementary Figure S7. **Non-tidal-TM case.** 12

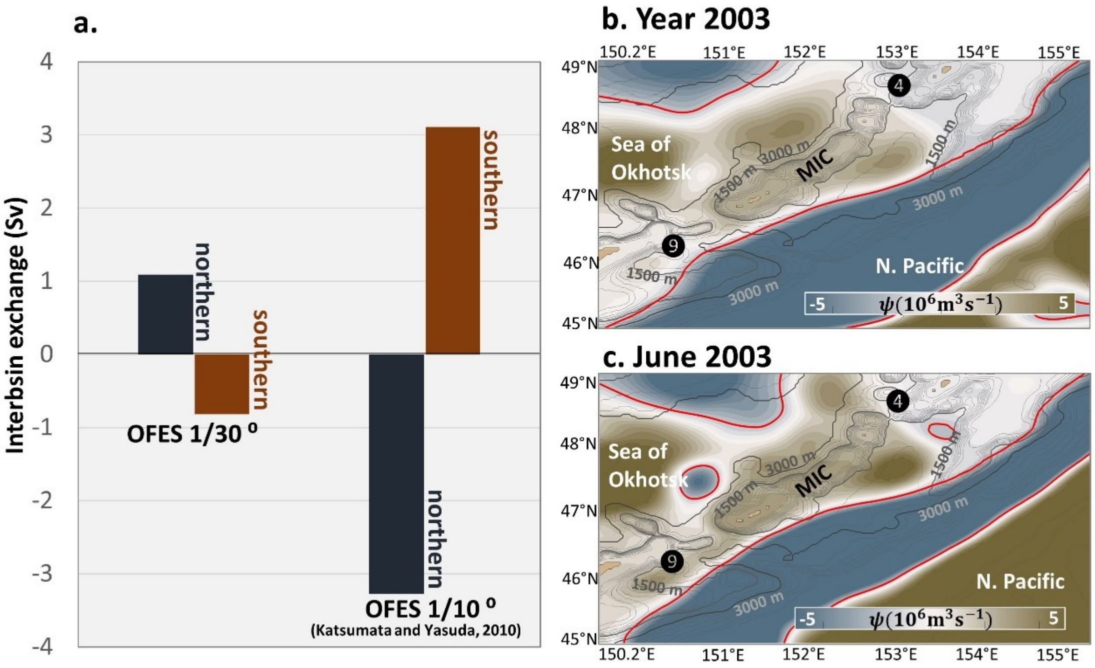

Supplementary Figure S1. **Results of the OFES 1/30° model.**

a. Annually-averaged volume transport in 2003 through two straits of the OFES 1/30° model[12,13] and OFES 1/10° model[7]. The deep-blue and brown boxes denote the annual-averaged transport through the Kruzensterna (nothern) and Bussol' (southern) Straits, respectively. The difference in exchange transport through both straits in the OFES 1/30° model is approximately three times smaller than that of the OFES 1/10° model. **b** Plan view of the annually-averaged streamfunction of the year 2003. **c** Plan view of the monthly-averaged streamfunction of June 2003. The grey contours denote the topography, where the contour interval is 150 m till 1500 m and continuously with 500 m intervals till 6000 m. The thick grey contours denote the 1500 m and 3000 m depth, respectively. The EKC in the OFES 1/30° model overleaps the MIC both in annually averaged and monthly-averaged instances as in the non-tidal case (Fig. 3**c**). The red contour is the streamfunction contour where $\psi =$

-1.5×10^6 m^3 s^{-1}. The streamfunction indicates that the transport crosses the MIC through its straits. Panels **b-c** are produced using Matlab (Matlab R2019b, https://www.mathworks.com/products/new_products/release2019b.html). The topography data is obtained from the OFES 1/30° model[12,13] (see the Methods).

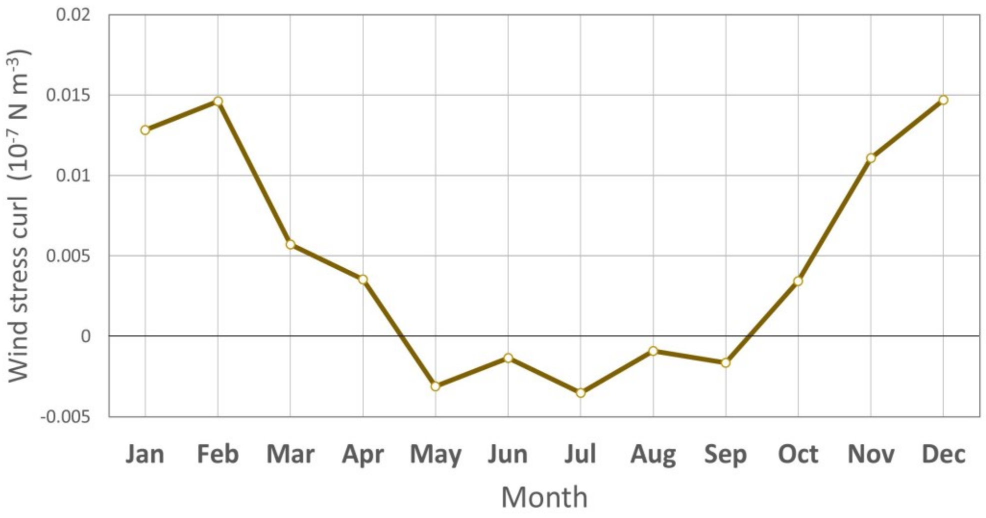

Supplementary Figure S2. **Local wind-stress-curl line integrated along a 1500 m contour surrounding the MIC.**

We used the monthly-averaged climatological wind stress from the Ocean Model Intercomparison Project (OMIP)[36,37], which was a 15-year daily reanalysis average from 1979 to 1993 obtained from the European Centre for Medium-Range Weather Forecasts[37] (see the Methods). As expected, the calculated wind stress curl along the 1500 m contour surrounding the MIC adhered to a circular theorem (see the Methods). The wind stress curl in summer (i.e., from May to September) is opposite from that in winter and its magnitude is also smaller in summer than in winter.

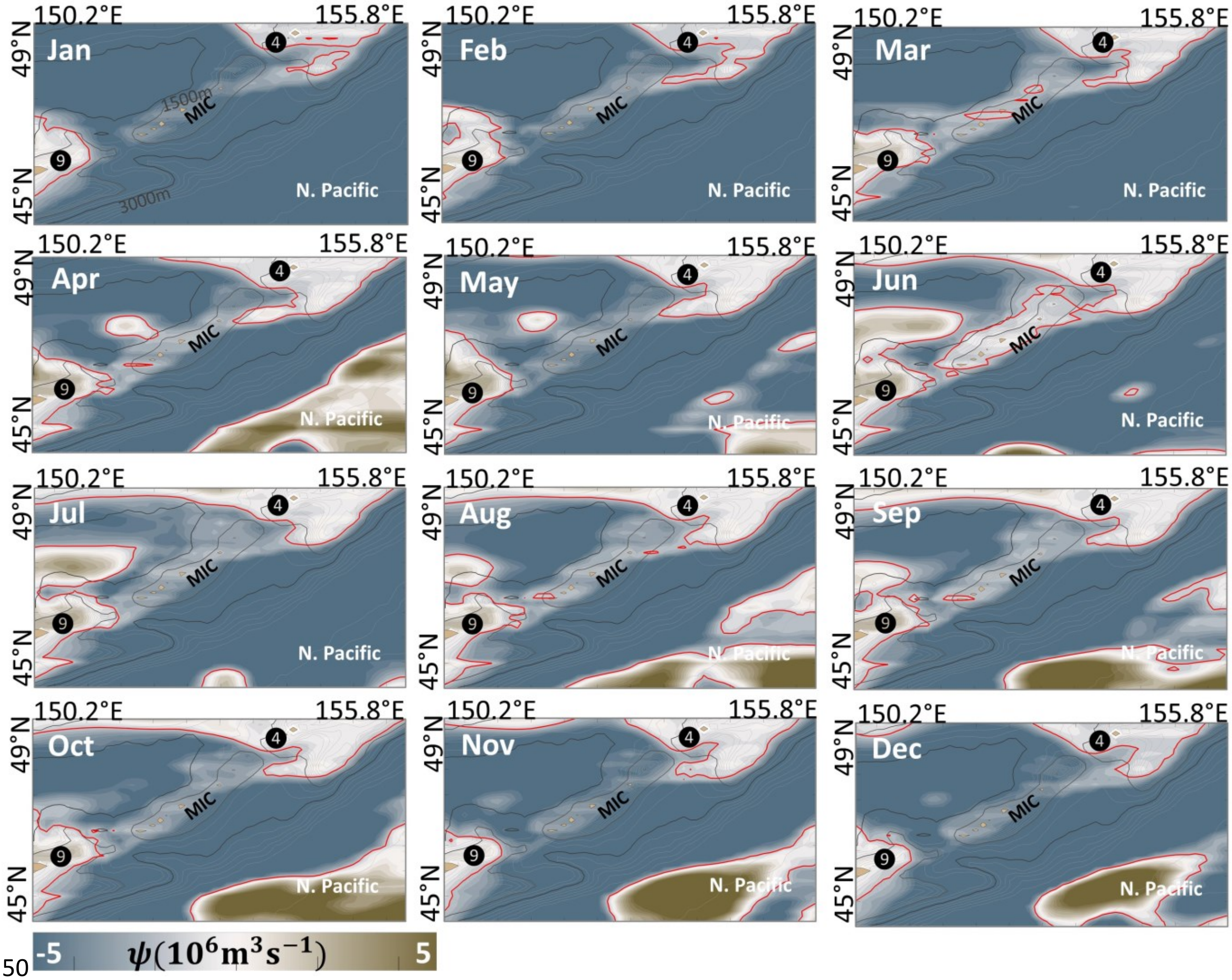

Supplementary Figure S3. Streamfunction of the tidal cases.

The shade denotes the streamfunction. The red contour denotes the streamfunction contour $\psi = -1.5 \times 10^6$ m^3 s^{-1}. The grey contours denote the topography, where the contour interval is 150 m till 1500 m and continuously with 500 m intervals till 6000 m. The thick grey contours denote the 1500 m and 3000 m depth, respectively. Monthly analyses are ordered from top to bottom and left to right. Although bifurcation points are formed every month, only the envelope of the streamfunction contour $\psi = -1.5 \times 10^6$ m^3 s^{-1} involves most of the

MIC in June representing the characteristic flow pattern, as illustrated in Fig. 1**b**. All panels are produced using Matlab (Matlab R2019b, https://www.mathworks.com/products/new_products/release2019b.html). The topography data is obtained from the COCO model (see the Methods).

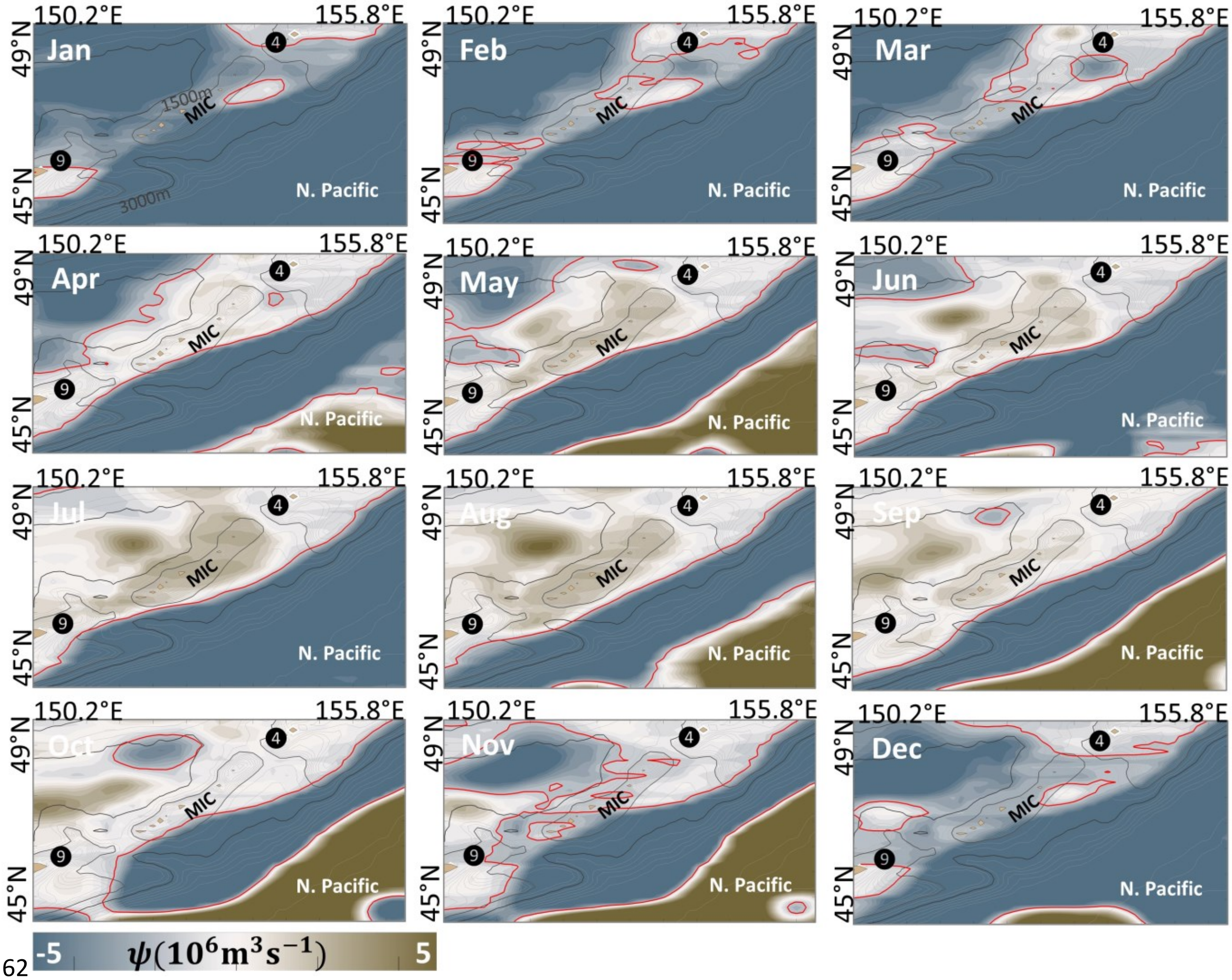

Supplementary Figure S4. Streamfunction of the non-tidal cases.

The shading in the illustrations represents the streamfunction. The red contour denotes the streamfunction contour $\psi = -1.5 \times 10^6$ m^3 s^{-1}. Topography contours are as same as Supplementary Fig. S3. The order of months is from top to bottom and left to right, respectively. From April to October, the streamfunction contours from the EKC blocks the interbasin exchange. The positive circulations above the MIC during the same period suggest that the cross-MIC vorticity fluxes are enormous as we described in the Methods. All panels

are produced using Matlab (Matlab R2019b, https://www.mathworks.com/products/new_products/release2019b.html). The topography data is obtained from the COCO model (see the Methods).

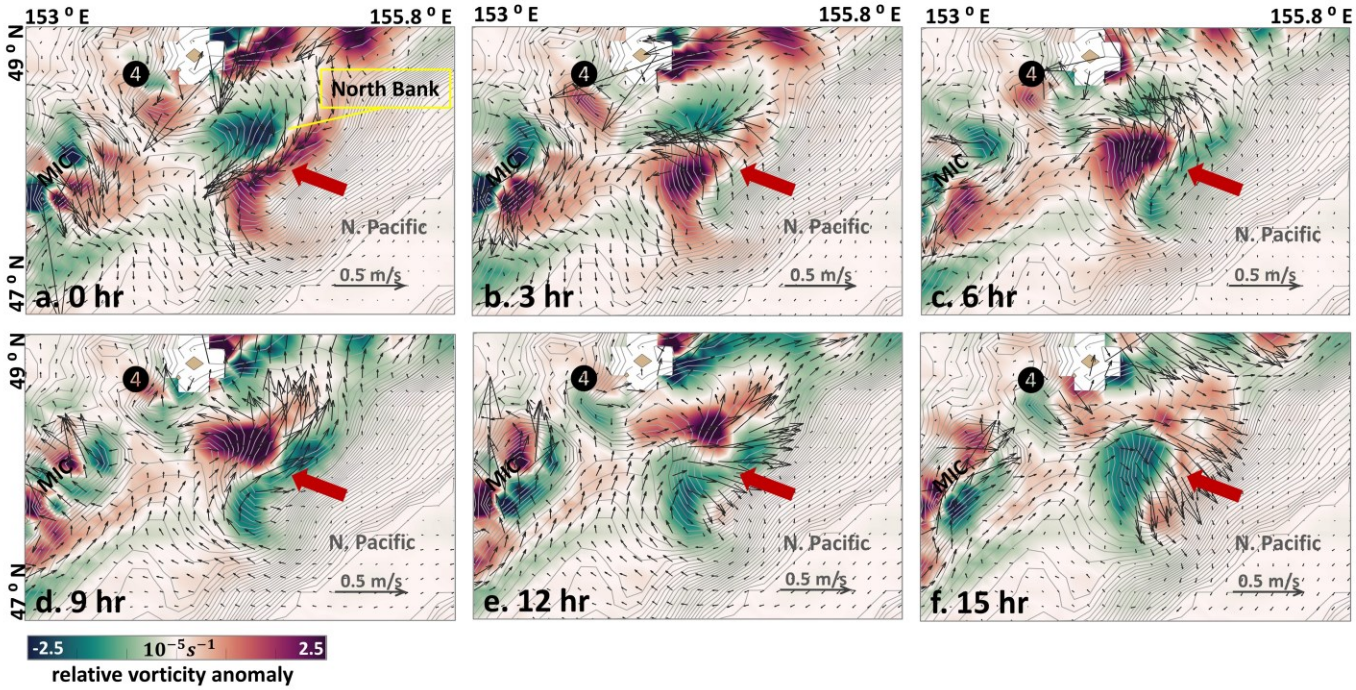

Supplementary Figure S5. Propagation of seamount trapped waves.

Snapshot of the plan view of the vertical averaged velocity anomaly (vector) and relative vorticity ($[\zeta] = \boldsymbol{k} \cdot \nabla \times [\boldsymbol{u}]$) anomaly (shade) of the tidal case in June at 3-hour time steps. The red arrows denote the propagation of the seamount trapped wave, which covers the entire range of the EKC. **a**. 0 hr. **b**. 3 hr. **c**. 6 hr. **d**. 9 hr. **e**. 12 hr. **f**. 15 hr. The seamount trapped wave propagates clockwise around the North Bank. All panels are produced using Matlab (Matlab R2019b, https://www.mathworks.com/products/new_products/release2019b.html). The topography data is obtained from the COCO model (see the Methods).

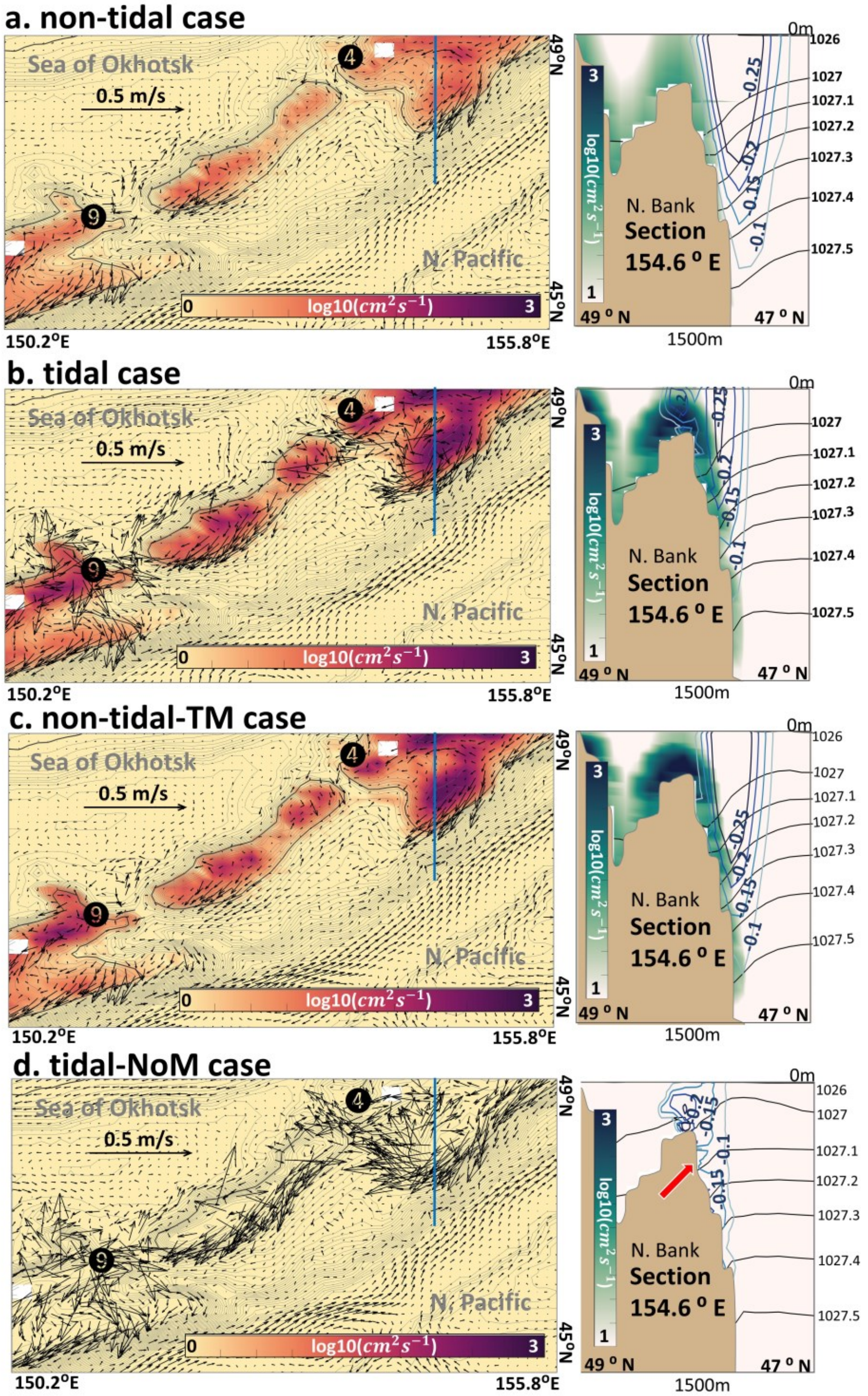

Supplementary Figure S6. **Bottom velocity and vertical diffusivity distribution in each case.**

Left panels: plan view of vertical diffusivity coefficient (shade, log 10 scale) and the bottom

velocity of the bottom friction layer (vector) in each case. Right panels: density structure (black contour), EKC core (blue contour), and the distribution of vertical diffusivity (shade) on the meridional section which extended northward from original Section A (blue line in left panels). **a**. non-tidal case. **b**. tidal case. **c**. non-tidal-TM case. **d**. tidal-NoM case. The red arrow indicates the area where the EKC sticks on the bottom surface without the vertical mixed layer. All panels are produced using Matlab (Matlab R2019b, https://www.mathworks.com/products/new_products/release2019b.html). The topography data is obtained from the COCO model (see the Methods).

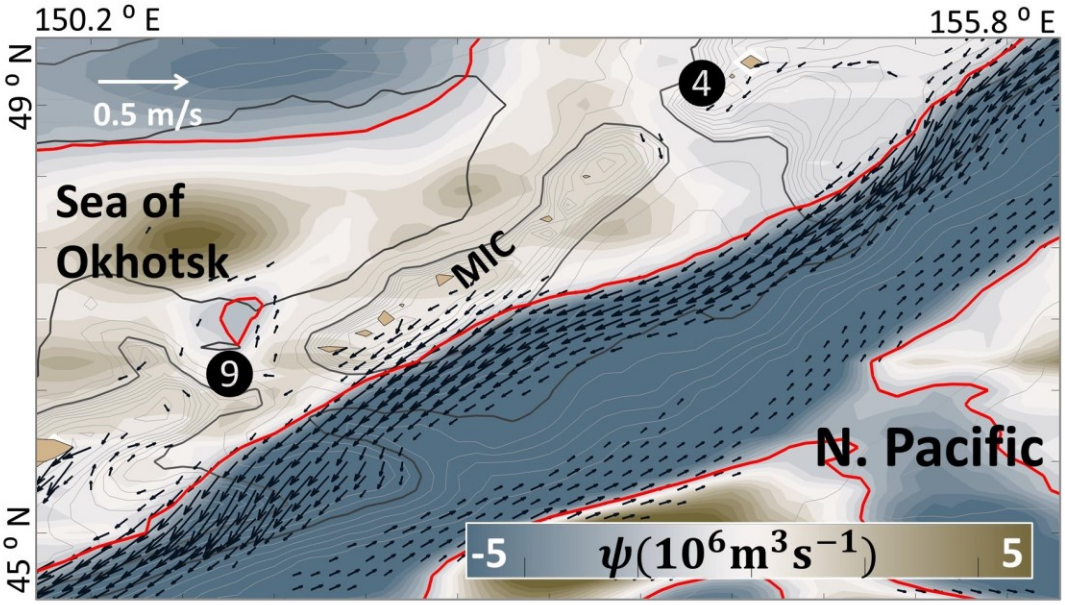

Supplementary Figure S7. **Non-tidal-TM case.**

Depth-averaged velocity (vectors in background figure) and streamfunction (shade in background figure) of the non-tidal-TM case. Only the vector which its' speed ($\sqrt{[u]^2 + [v]^2}$) over 0.05 m s^{-1} is shown. Topography contours are as same as Supplementary Fig. S3.The red contour is the streamfunction contour $\psi = -1.5 \times 10^6$ m^3 s^{-1}. The streamfunction shows that the non-tidal-TM case has a similar structure to the non-tidal case. This figure is produced using Matlab (Matlab R2019b, https://www.mathworks.com/products/new_products/release2019b.html). The topography data is obtained from the COCO model (see the Methods).
